# Analysis of Chemosensory Genes in Full and Hungry Adults of *Arma chinensis* (Pentatomidae) Through Antennal Transcriptome

**DOI:** 10.3389/fphys.2020.588291

**Published:** 2020-11-06

**Authors:** Shaolong Wu, Wan Deng, Mi Li, Yansong Xiao, Jiaying Li, Kai Teng, Zhipeng Xiao, Xiaohong Li, Zhicheng Zhou, Youzhi Li

**Affiliations:** ^1^College of Plant Protection, Hunan Agricultural University, Changsha, China; ^2^Hunan Province Tobacco Company, Changsha, China; ^3^Hunan Academy of Forestry, Changsha, China; ^4^College of Urban and Rural Construction, Shaoyang University, Shaoyang, China

**Keywords:** *Arma chinensis*, chemoreception genes, transcriptome, gender, olfactory system

## Abstract

The predatory insect *Arma chinensis* (Hemiptera: Pentatomidae) is widely distributed in China, where it is also used to control many agricultural and forest pests. The chemosensory genes expressed in its antennae play crucial roles in food-seeking and mating behaviors. To better understand the olfaction of *A. chinensis* antennae, we identified the genes related to food-seeking and mating. Sequencing of the antennal transcriptomes of full and hungry male and female *A. chinensis* revealed 38 odorant-binding proteins (OBPs), 1 chemosensory protein (CSP), 1 Niemann–Pick C2 protein (NPC2), 3 odorant receptors (ORs), 12 ionotropic receptors (IRs), 2 gustatory receptors (GRs), and 3 sensory neuron membrane proteins (SNMPs). These results were used to construct phylogenetic trees. A quantitative real-time PCR (qRT-PCR) analysis showed that the relative transcript levels of *AchiGR1*, *AchiGR2*, and *AchiOBP28* were higher in female than in male antennae in both full and hungry insects, but that the expression of *AchiOBP13* and *AchiOBP16* was higher only in full *A. chinensis* females. Thus, the latter genes may encode proteins involved in oviposition selection behavior. *AchiGRs* (*1* and *2*), *AchiIR6*, and *AchiOBPs* (*6–8*, *12*, *20–22*, *28*, and *34*) were highly expressed only in the antennae of full males, indicating the participation of these genes in mate-searching or male pheromone recognition. The expression of *AchiOBP31* in the antennae of starved males, *AchiOBPs* (*15*, *18*, and *29*) in the antennae of starved females, and *AchiOBPs* (*3*, *4*, and *24*) in the antennae of starved males and females suggested that these genes encode food-seeking functions. Our identification of chemosensory genes in *A. chinensis* antennae and their differential expression in full and hungry insects provides the basis for further functional studies on the chemoreception system of *A. chinensis* and the sex hormones of predatory insects.

## Introduction

In insects, the olfactory system plays crucial roles in predation, mating, oviposition, and survival (Ji et al., 2013; Leal, 2013; Zhou et al., 2010). Antennae are the main olfactory organ in the chemosensory system of insects and through complex biochemical reactions ensure the precise conduction of chemical signals (Vogt et al., 1985; Benton et al., 2009). The major olfactory proteins include odorant-binding proteins (OBPs), chemosensory proteins (CSPs), odorant receptors (ORs), gustatory receptors (GRs), ionotropic receptors (IRs), Niemann–Pick C2 proteins (NPC2s), and sensory neuron membrane proteins (SNMPs) (Korsching, 2002; Rützler and Zwiebel, 2005; Pelosi et al., 2006; Sato and Touhara, 2008; Leal, 2013). In the insect olfactory system, chemical signals perceived by the antennae are converted into electrical signals, which are then transmitted to the brain and induce the intended behaviors (Korsching, 2002; Touhara and Vosshall, 2009; Jia et al., 2016; Liu et al., 2017). When hydrophobic odorants in the environment enter the antennae, they bind to OBPs and CSPs and are then transported through the sensillar lymph to activate membrane-bound ORs (Cao et al., 2014; Gu et al., 2014). Subsequent binding of the odorants to chemosensory receptors (ORs, IRs, and GRs) results in the generation of electrical signals. Inactivation of the signal is achieved by odorant degrading enzymes (ODEs), which inhibit signal accumulation and ensure that insects are able to react quickly to ever-changing odorants in their environment (Korsching, 2002; Rützler and Zwiebel, 2005; Jia et al., 2018).

Chemical cues alert predator insects that feed on herbivorous insects to the presence of their prey (Frago et al., 2017). For example, *Microplitis mediator* is attracted by herbivore-induced plant volatiles (Geneau et al., 2013; Patt and Rohrig, 2017), and *Aphidius ervi* utilizes the synomones produced by herbivorous insects as signaling the presence of a host (Glinwood et al., 1999a,b). The predatory insect *Arma chinensis*, the focus of this study, is widely distributed in China (Rider and Zheng, 2002), where it is also used to effectively control many agricultural and forest pests, such as coleopteran, hemipteran, hymenopteran, and lepidopteran insects (Gao et al., 2011; Zou et al., 2012). *A. chinensis* largely relies on pheromone isomerism to search for food, with its antennae serving as key allelopathic organs. However, the number of chemoreceptor genes expressed in *A. chinensis* antennae and the distinction between genes related to the search for food and the detection of pheromone isomerism are unclear. Insights gained from an elucidation of the olfactory system of *A. chinensis* can be applied to increase the efficiency of this predator insect in bio-control (Wang et al., 2017, 2018).

In this study, next-generation sequencing was used to identify the chemoreception genes of *A. chinensis* antennae and the sex specificity of those genes in full and hungry male and female *A. chinensis* (Wang et al., 2017, 2018), and we assumed that the chemosensory genes would be different between full and hungry adults of *A. chinensis*’ antennae. From the datasets of the *A. chinensis* antennal transcriptome, 38 OBP, 1 CSP, 1 NPC2, 3 OR, 12 IR, 2 GR, and 3 SNMP genes were identified. Sequence information was used to construct phylogenetic trees and thereby infer the evolutionary relationships of *A. chinensis* with other insects. In addition, the relative transcript levels of these chemoreception genes were determined using quantitative real-time PCR (qRT-PCR).

## Materials and Methods

### Insect Rearing and Sample Collection

*Arma chinensis* collected in Langfang, Hebei Province, China in 2018 was fed on Chinese oak silk moth pupae purchased from a supermarket in Liaoning. The rearing of *A. chinensis* was as described in a previous study (Pan et al., 2019). The insects were divided into four treatment groups: full females, full males, hungry females, and hungry males. In the full groups, adult male and female *A. chinensis* were fed sufficient food for 3 days, whereas in the hungry treatments, food was supplied for 2 days after which the insects were starved for 24 h. After 3 days, the antennae of all adult males and females were removed, frozen in liquid nitrogen, and stored at −80°C until required. Each treatment consisted of three biological replicates, and each biological replicate contained 100 pairs of adult antennae.

### Total RNA Extraction

Total RNA was extracted from tissues using TRIzol (Invitrogen, Carlsbad, CA, United States) according to the manufacturer’s instructions. Sixty mg of tissue was ground into powder in liquid nitrogen in 2 ml tubes and homogenized for 2 min. The tubes were then rested horizontally for 5 min. After centrifugation at 12,000 × *g* for 5 min at 4°C, the supernatants were transferred into new centrifugal tubes (Eppendorf) containing 0.3 ml of chloroform/isoamyl alcohol (24:1). The mixture was shaken vigorously for 15 s and then centrifuged as in the preceding step. From each tube, the upper aqueous phase, containing the RNA, was transferred into a new tube, and an equal volume of isopropyl alcohol was added. After centrifugation at 13,600 rpm for 20 min at 4°C, the supernatants were discarded, and the RNA pellets were washed twice with 1 ml of 75% ethanol. The suspensions were centrifuged at 13,600 rpm for 3 min at 4°C, and the resulting pellets were air-dried for 5–10 min in a biosafety cabinet. Finally, the RNA was dissolved in 25–100 μl of DEPC-treated water. Total RNA was evaluated qualitatively and quantitatively using a NanoDrop and Agilent 2100 Bioanalyzer (Thermo Fisher Scientific, MA, United States).

### mRNA Library Construction

mRNA was purified using oligo(dT)-attached magnetic beads and fragmented into small pieces using fragment buffer (containing Mg^2+^) at the appropriate temperature. A first-strand cDNA was generated using random hexamer-primed reverse transcription, followed by a second-strand cDNA synthesis. The cDNA ends were repaired by incubating the fragments with A-Tailing Mix and RNA Index Adapters. The cDNAs were then PCR amplified. The PCR products were purified using AMPure XP beads, dissolved in elution buffer (EB) solution, and then validated on an Agilent Technologies 2100 Bioanalyzer for quality control. The double-stranded PCR products were heat-denatured and circularized using the splint oligo sequence, with the resulting single-stranded circular DNAs comprising the final library. DNA nanoballs (DNBs) containing >300 copies of the circular DNAs were obtained by amplification of the library with phi29. The DNBs were loaded into a patterned nanoarray, and paired-end 100-base reads were generated on a BGIseq500 platform (BGI-Shenzhen, China).

### Bioinformatics Analysis

Raw reads were filtered using SOAPnuke (v1.4.0) (Chen et al., 2018) by removing reads containing adaptors, poly-N, or low quality. The clean reads were then assembled using Trinity (v2.0.6) (Grabherr et al., 2011), followed by Tgicl (v2.0.6) (Pertea et al., 2003) to cluster the reads and eliminate redundant data in the assembled transcripts.

The clean reads were mapped to the assembled unique genes using Bowtie2 (v2.2.5) (Langmead and Salzberg, 2012), and the expression levels of those genes were calculated using RSEM (v1.2.8) (Dewey and Li, 2011). The results were normalized to fragments per kilo base of transcript per million mapped reads (FPKM). The genes were functionally annotated by mapping them to different databases [nucleotide sequences (NT), non-redundant protein (NR), euKaryotic Ortholog Groups (KOG), Kyoto Encyclopedia of Genes and Genomes (KEGG)] using the BLAST software (v2.2.23) (Miller et al., 2008). Gene Ontology (GO) annotation was performed using Blast2GO (v 2.5.0) with NR annotation. DEseq2 (Love et al., 2014) was used to detect differentially expressed genes (DEGs), and DEGs with a fold change >2 or < −2 and an adjusted *P*-value ≤ 0.001 were considered to be significant. GO and KEGG enrichment analyses were performed using Phyper, a function of R. The significance levels of the terms and pathways were corrected based on a *Q*-value with a rigorous threshold (<0.05).

### Identification of Candidate Chemoreception Genes and Phylogenetic Analysis

The tBLASTn program was used to identify candidate unigenes encoding putative OBPs, CSPs, ORs, IRs, and SNMPs in *A. chinensis* using available sequences of these proteins from Pentatomidae species. All candidate genes were manually checked using BLASTX against GenBank non-redundant (nr) protein database at the National Center for Biotechnology Information (NCBI). ORF Finder was used to predict open reading frames (ORFs), signalP 5.0 to predict OBP and CSP signal peptides, and the TMHMM Server v. 2.0 to predict OR and IR transmembrane domains (TMDs). The differential expression of *A. chinensis* chemoreception genes in the antennae of full and hungry adult insects was determined using FPKM, as described by Qiu et al. (2018). MEGA-X and Clustal W were used to align amino acid sequences, and maximum likelihoods were used to construct the phylogenetic trees (JTT model, 1,000 bootstrap replications) (Larkin et al., 2007; Price et al., 2010). The phylogenetic trees were mainly based on the amino acid sequences of *A. chinensis* and other insect species. Those species used to construct the phylogenetic tree of different chemosensory genes are presented in Supplementary Table 1.

## qRT-PCR and Statistical Analysis

We used qRT-PCR with three replicates for each treatment to verify the expression of candidate *A. chinensis* chemosensory genes. cDNA was synthesized from total RNA using a PrimeScript RT reagent kit with gDNA eraser (perfect real time) (Takara, Dalian, China) according to the manufacturer’s instructions, as described by Qiu et al. (2018). qRT-PCR primers were designed online by the NCBI’s profile server^1^ and the 18S rRNA genes as reference genes. The reaction conditions were as follows: (1) 95°C for 30 s, (2) 95°C for 5 s, (3) 60°C for 30 s, and (4) 95°C for 5 s, 40 cycles in a CFX96 machine (Bio-Rad, Japan). Melting curve analysis was performed from 55 to 95°C to determine the specificity of qRT-PCR primers. Gene expression profiles were analyzed using the 2^–ΔΔCt^ method (Livak and Schmittgen, 2001). The means and variances of the treatments were analyzed in a one-way ANOVA using R v.3.3.3 (R Core Team, 2017).

## Results

### Analysis of the *A. chinensis* Transcriptome

From the antennal transcriptomes of females, 49.19 million (full female-1), 49.19 million (full female-2), 49.19 million (full female-3), 49.19 million (hungry female-1), 47.43 million (hungry female-2), and 49.19 million (hungry female-3) raw reads were obtained, and from the antennal transcriptomes of males, 49.19 million (full male-1), 47.18 million (full male-2), 47.43 million (full male-3), 49.19 million (hungry male-1), 47.43 million (hungry male-2), and 47.43 million (hungry male-3) raw reads were obtained (Supplementary Table 2). Filtering yielded 44.37 million (full female-1), 44.21 million (full female-2), 44.13 million (full female-3), 44.18 million (hungry female-1), 42.87 million (hungry female-2), 44.21 million (hungry female-3), 43.64 million (full male-1), 42.95 million (full male-2), 43.11 million (full male-3), 44.89 million (hungry male-1), 43.05 million (hungry male-2), and 42.88 million (hungry male-3) clean reads (Supplementary Table 2). These clean reads generated 34,407 unigenes with total, mean, N50, N70, and N90 lengths of 40,468,637, 1,176, 1,932, 1,224, and 510 bp, respectively (Supplementary Table 3).

### Functional Annotation of the *A. chinensis* Antennal Unigenes

**FIGURE 1:**
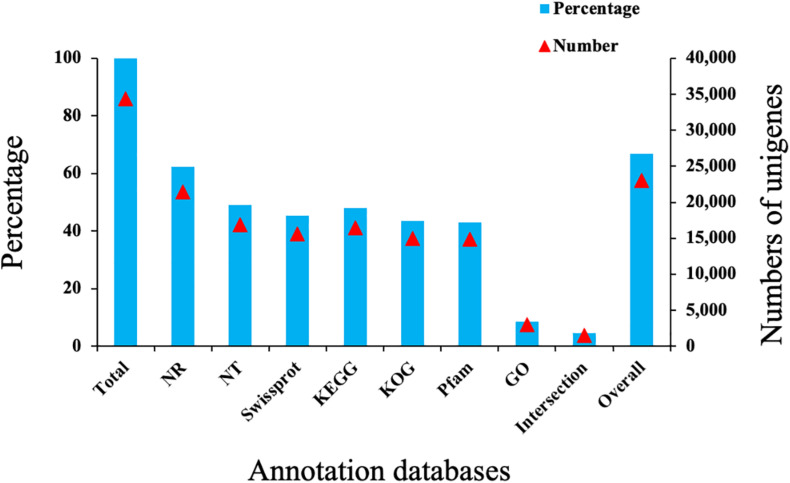
Summary of the unigene annotations in the *Arma chinensis* antennal transcriptome. Total, all annotation unigenes; NR, non-redundant protein; NT, nucleotide sequences; Swiss-prot, a manually annotated and reviewed protein sequence database; KEGG, Kyoto Encyclopedia of Genes and Genomes; KOG, euKaryotic Ortholog Groups; Pfam, protein family; GO, Gene Ontology; Intersection, unigenes annotated in all databases; Overall, unigenes annotated in at least one of the databases.

**FIGURE 2:**
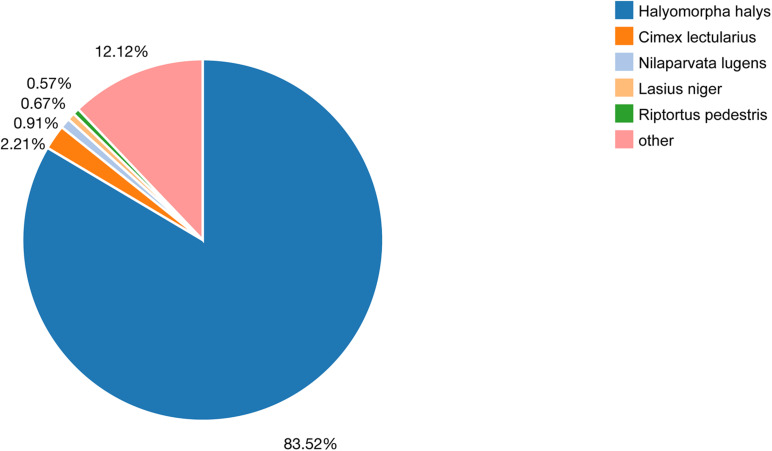
Species distribution of the unigenes within the *A. chinensis* antennal transcriptome based on the results of a BLASTX search. Different colors represent different species.

**FIGURE 3:**
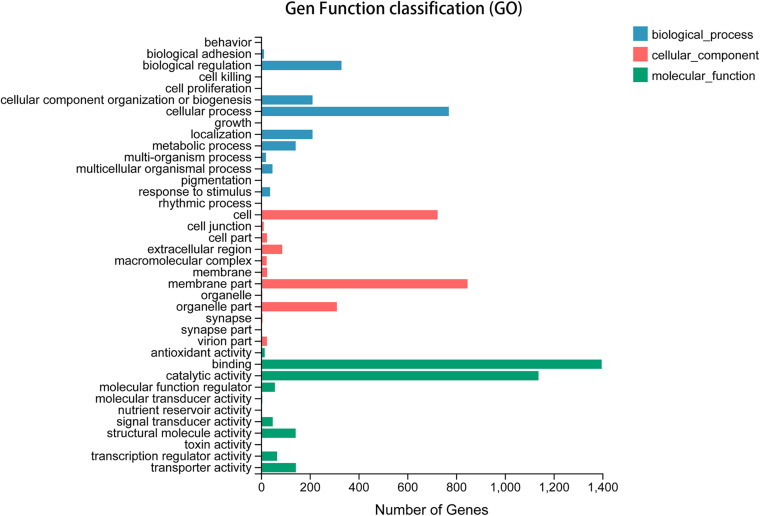
Gene ontology classifications of *A. chinensis* antennal transcriptome unigenes. The abscissa *x*-axis denotes the number of genes in the category.

By searching the referenced databases, we were able to annotate 23,045 (66.98%) unigenes, with 1,540 (4.48%) unigenes annotated in all databases. The largest numbers of unigene annotations were achieved using the NR database (21,442, 62.32%), followed by the NT, KEGG, KOG, and Pfam databases (43.11–49.03%) (Figure 1). Based on a BLASTX homology search against the NCBI-NR database, the best match of *A. chinensis* sequences was with the sequences of *Halyomorpha halys* (83.52%), followed by *Cimex lectularius* (2.21%), *Nilaparvata lugens* (0.91%), and other species (12.12%) (Figure 2). In the GO analysis, 8.57% (2,949) of the unigenes from the *A. chinensis* antennal transcriptome were assigned to three main functions: “biological process,” “cellular component,” and “molecular function” (Figure 3). Of these, the most prevalent term within “biological process” was cellular processes (1,790, 43.17%), followed by biological adhesion (330, 18.44%), localization (211, 11.79%), and cellular component organization or biogenesis (211, 11.79%). Within “cellular component,” membrane part (847, 40.553%) was the most prevalent, followed by cell (724, 34.66%), and within “molecular function” binding (1,397, 46.37%) was the most prevalent term, followed by catalytic activity (1,138, 37.77%) (Figure 3).

### Identification of Candidate OBPs

All 38 OBP genes (named *AchiOBP1–38*) identified from the dataset of the *A. chinensis* antennal transcriptome had full-length ORFs with signal peptides. Their amino acid sequences and the BLASTX results are presented in Supplementary Tables 4, 5. Based on a homology search in NCBI, all AchiOBP sequences were best matched to the OBPs of known Pentatomidae, with >90% sequence identities for five pairs of OBPs, AchiOBP11 and NvirOBP4 (97%), AchiOBP1 and NvirOBP9 (94%), AchiOBP2 and NvirOBP9 (94%), AchiOBP28 and HhalOBP1 (93%), and AchiOBP37 and NvirOBP14 (90%). Only AchiOBP12 and AchiOBP19 had sequence identities of <50%; for the other 31 pairs, the sequence identities ranged from 51 to 89% (Supplementary Table 5).

**FIGURE 4:**
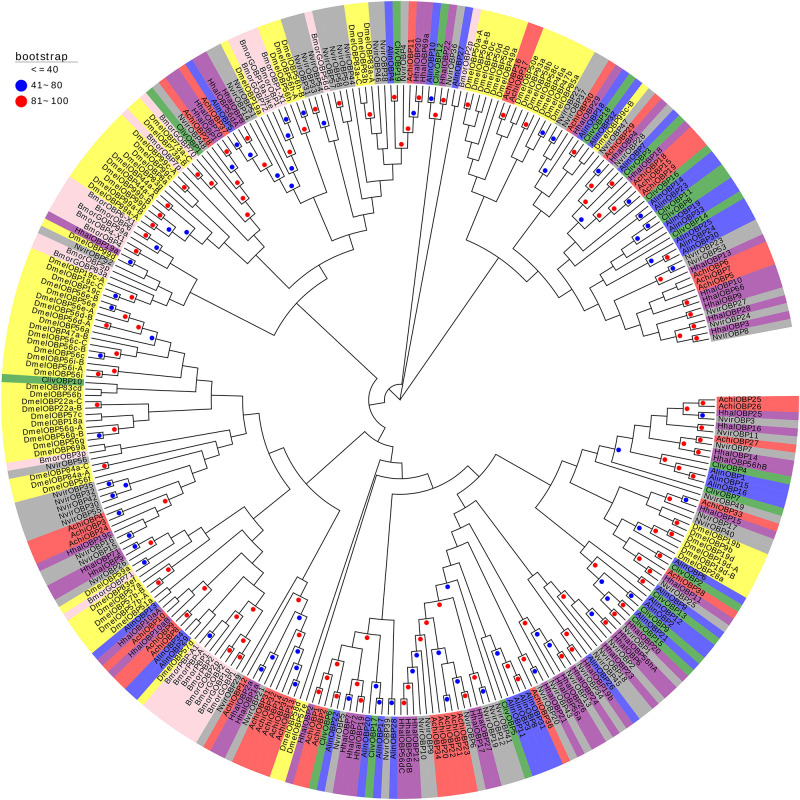
Phylogenetic analysis of the OBPs (odorant-binding proteins) from seven insect species: *A. chinensis* (Achi, red), *D. melanogaster* (Dmel, yellow), *C. lividipennis* (Cliv, green), *A. lineolatus* (Alin, blue), *H. halys* (Hhal, purple), *N. viridula* (Nvir, gray), and *B. mori* (Bmor, beige). Red circles mean bootstrap values are 81–100, blue circles mean bootstrap values are 41–80, and hide bootstrap values are less than 41.

The phylogenetic tree constructed based on the OBPs of *A. chinensis* and six other species, including four Hemiptera (*H. halys*, *Nezara viridula*, *Cyrtorhinus lividipennis*, *Adelphocoris lineolatus*), one Diptera (*Drosophila melanogaster*), and one Lepidoptera (*Bombyx mori*), positioned the AchiOBPs along several different branches, with all AchiOBPs closed to NvirOBPs and HhalOBPs (Figure 4). Most of the AchiOBPs were on the same branch as NvirOBPs or HhalOBPs, including AchiOBP27 and NvirOBP7, AchiOBP30 and HhalOBP29, AchiOBP29 and HhalOBP4, and AchiOBP33 and HhalOBP15 (Figure 4).

### Identification of Candidate CSP and NPC2

One CSP gene (*AchiCSP1*) and NPC2 gene (*AchiNPC2*) were identified from the dataset of the *A. chinensis* antennal transcriptome. The protein sequence and BLASTX results of AchiCSP and AchiNPC2 are presented in Supplementary Tables 4, 5. *AchiNPC2* but not *AchiCSP1* had a full-length ORF. The BLASTX results of CSP and NPC2 are presented in Supplementary Table 4. AchiCSP1 showed 54% sequence identity to TeleCSP2 and 48% sequence identity to NvirCSP9. AchiNPC2 showed 83% sequence identity to HhalNCP2 and 42% sequence identity to ClivNPC2, AlinNPC2, and HhalNPC2 (Supplementary Table 5).

A phylogenetic tree was constructed to determine the relationships of AchiCSP to the equivalent proteins of other five species, including three Hemiptera (*N. viridula*, *C. lividipennis*, *A. lineolatus*), one Diptera (*D. melanogaster*), and one Lepidoptera (*B. mori*). Meanwhile, another phylogenetic tree was constructed to determine the relationships of AchiNPC2 to the equivalent proteins of other eight Hemiptera, Hymenoptera, Diptera, and Lepidoptera species, including three Hemiptera (*H. halys*, *C. lividipennis*, *A. lineolatus*), three Hymenoptera (*M. mediator*, *Macrocentrus cingulum*, *Camponotus japonicus*), one Diptera (*D. melanogaster*), and one Lepidoptera (*Operophtera brumata*) species.

**FIGURE 5:**
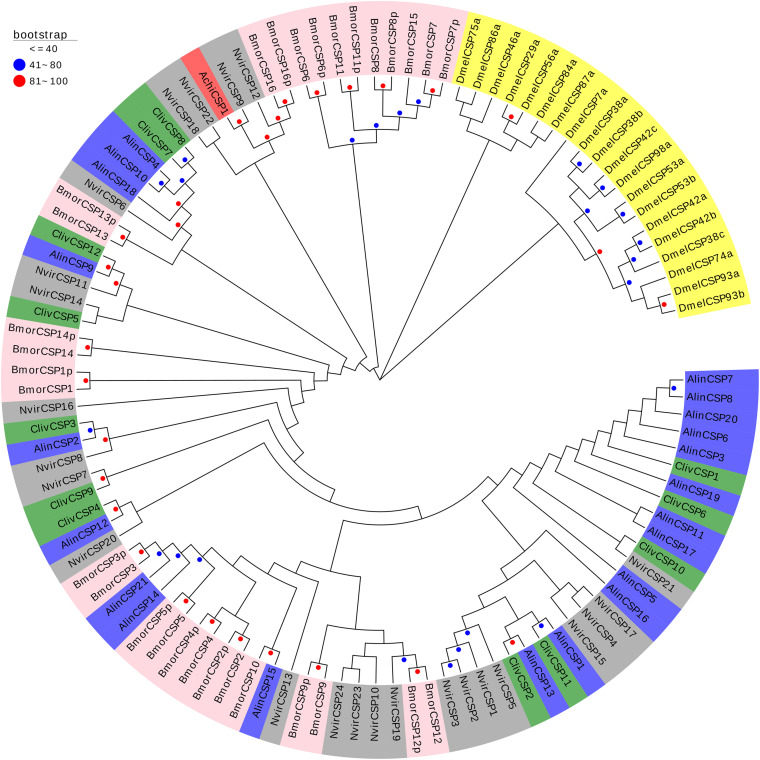
Phylogenetic analysis of the CSPs (chemosensory proteins) from six insect species: *A. chinensis* (Achi, red), *D. melanogaster* (Dmel, yellow), *C. lividipennis* (Cliv, green), *A. lineolatus* (Alin, blue), *N. viridula* (Nvir, gray), and *B. mori* (Bmor, beige). Red circles mean bootstrap values are 81–100, blue circles mean bootstrap values are 41–80, and hide bootstrap values are less than 41.

**FIGURE 6:**
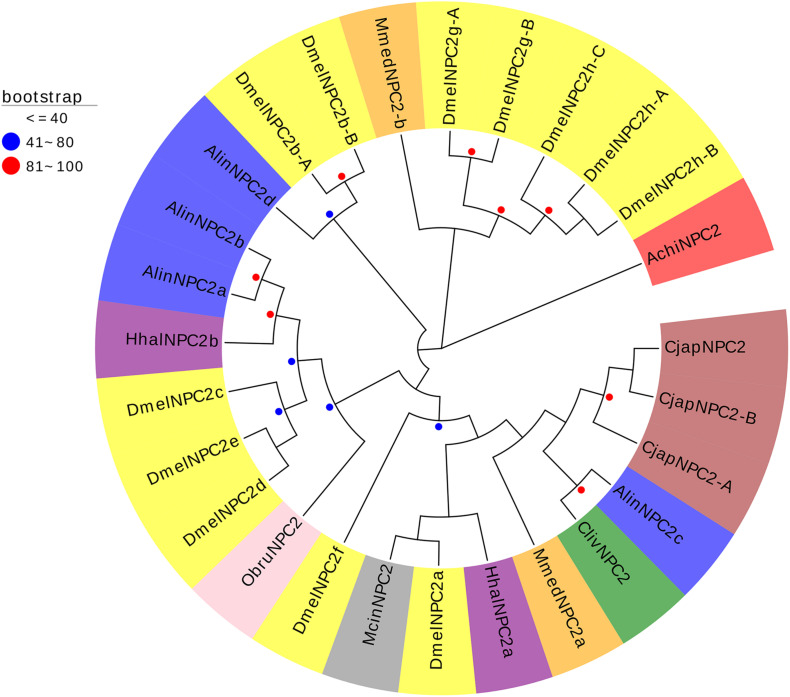
Phylogenetic analysis of the NPC2s (Niemann–Pick C2 proteins) from nine insect species: *A. chinensis* (Achi, red), *D. melanogaster* (Dmel, yellow), *H. halys* (Hhal, purple), *C. lividipennis* (Cliv, green), *A. lineolatus* (Alin, blue), *M. cingulum* (Mcin, gray), *M. mediator* (Mmed, orange), *C. japonicus* (Cjap, brown), and *O. brumata* (Obru, beige). Red circles mean bootstrap values are 81–100, blue circles mean bootstrap values are 41–80, and hide bootstrap values are less than 41.

The CSPs phylogenetic tree indicated that AchiCSP1 was in the same branch as NvirCSPs9 and on a subbranch with NvirCSPs12, BmorCSP16, and BmorCSP16p (Figure 5). The AchiNPC2 phylogenetic tree placed AchiNPC2 was in the same branch with DmelNPC2g (A and B) and DmelNPC2h (A,B, and C) (Figure 6).

### Identification of Chemoreceptor Genes

Three candidate ORs, 12 candidate IRs, and 2 GRs were identified from the dataset of the *A. chinensis* antennal transcriptome. The protein sequences and BLASTX results of AchiIRs, AchiORs, and AchiGRs are presented in Supplementary Tables 4, 5. *AchiOR2* and *AchiOR3* were represented by full-length ORFs. AchiOR1, AchiOR2, and AchiOR3 had 28, 30, and 47% sequence identity with HhalOR43a, HhalOR4, and HhalOR4, respectively. All AchiIRs were predicted to have full-length ORFs with a least one TMD, and all AchiIRs showed >89% sequence identity with other HhalIRs (Supplementary Table 5). AchiGR2 shared 64% sequence identity with HhalGR2a, whereas there was no sequence identity between AchiGR1 and the sequences of any other species in the NCBI dataset.

**FIGURE 7:**
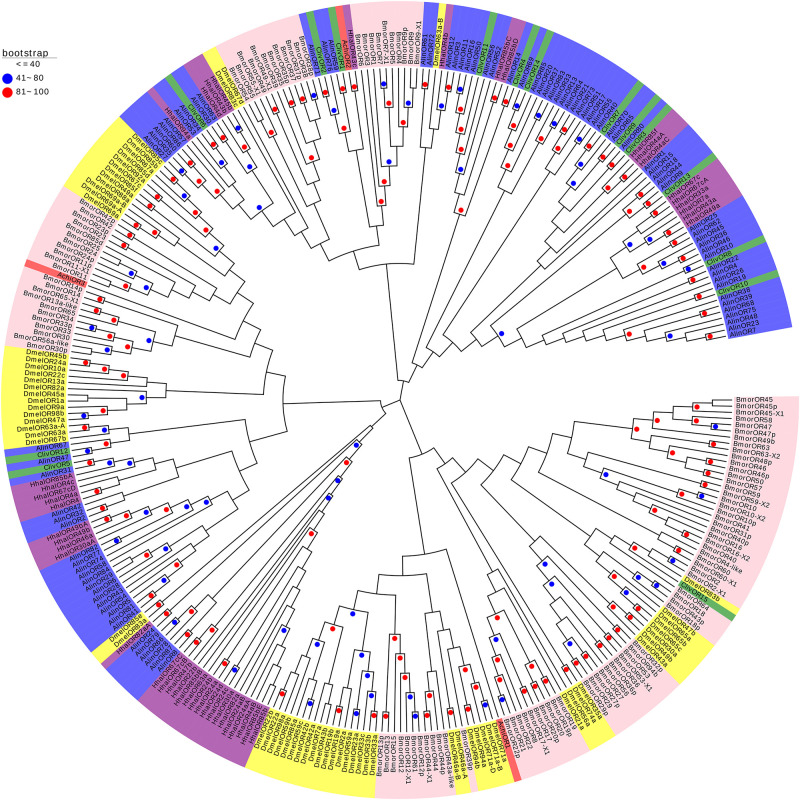
Phylogenetic analysis of the ORs (odorant receptors) from six insect species: *A. chinensis* (Achi, red), *D. melanogaster* (Dmel, yellow), *H. halys* (Hhal, purple), *C. lividipennis* (Cliv, green), *A. lineolatus* (Alin, blue), and *B. mori* (Bmor, beige). Red circles mean bootstrap values are 81–100, blue circles mean bootstrap values are 41–80, and hide bootstrap values are less than 41.

**FIGURE 8:**
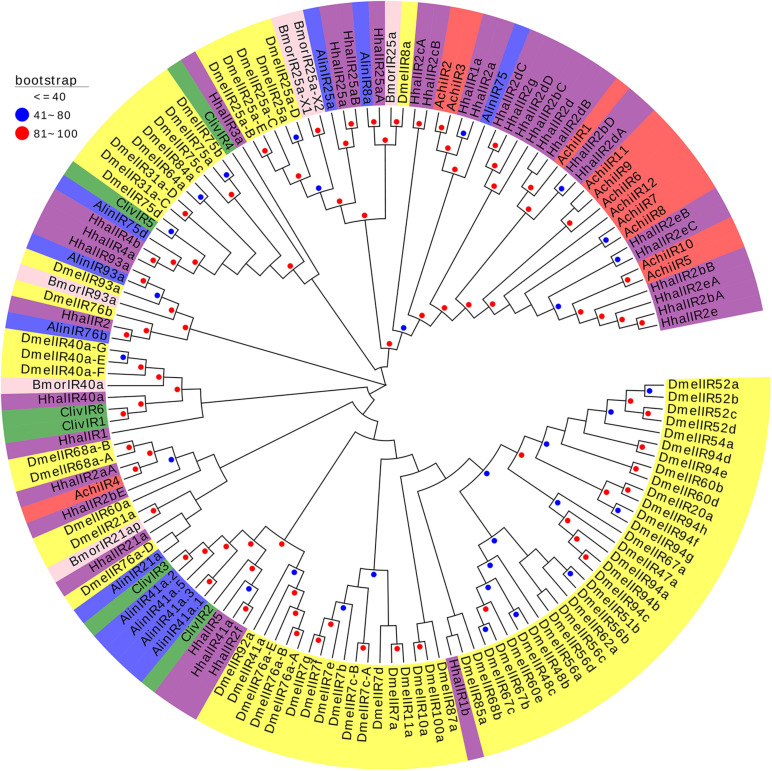
Phylogenetic analysis of the IRs (ionotropic receptors) from six insect species: *A. chinensis* (Achi, red), *D. melanogaster* (Dmel, yellow), *H. halys* (Hhal, purple), *C. lividipennis* (Cliv, green), *A. lineolatus* (Alin, blue), and *B. mori* (Bmor, beige). Red circles mean bootstrap values are 81–100, blue circles mean bootstrap values are 41–80, and hide bootstrap values are less than 41.

**FIGURE 9:**
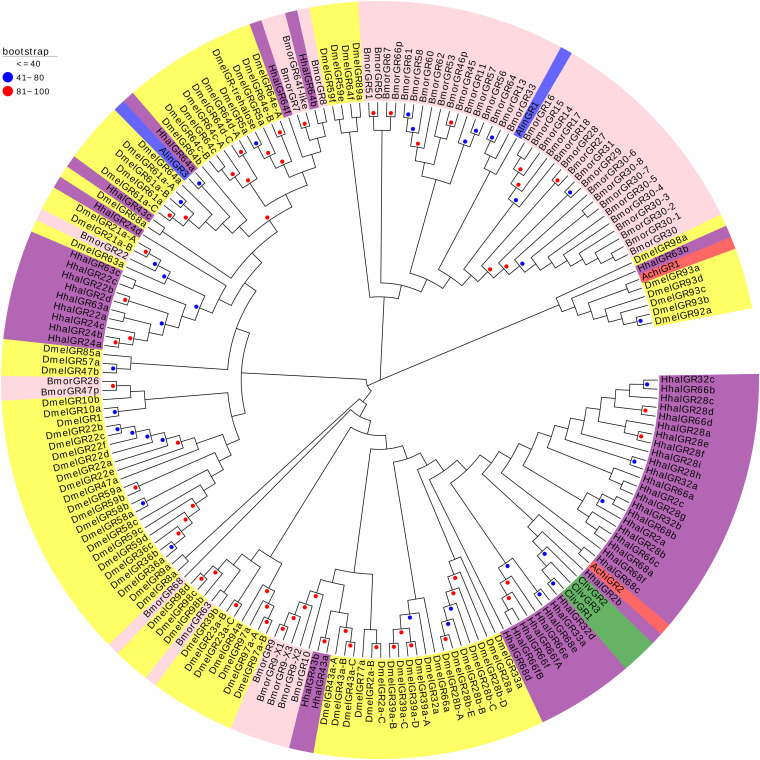
Phylogenetic analysis of the GRs (gustatory receptors) from six hemipteran insect species: *A. chinensis* (Achi, red), *D. melanogaster* (Dmel, yellow), *H. halys* (Hhal, purple), *C. lividipennis* (Cliv, green), *A. lineolatus* (Alin, blue), and *B. mori* (Bmor, beige). Red circles mean bootstrap values are 81–100, blue circles mean bootstrap values are 41–80, and hide bootstrap values are less than 41.

Three phylogenetic trees were constructed to better understand the relationships of AchiORs, AchiIRs, and AchiGRs with the respective ORs, IRs, and GRs of five other species: three Hemiptera (*H. halys*, *C. lividipennis*, *A. lineolatus*), one Diptera (*D. melanogaster*), and one Lepidoptera (*B. mori*) species. AchiORs were located on three different branches; AchiOR2 was on the same branch with HhalOR43c, and AchiOR1 and AchiOR3 were near to BmorORs and DmelORs (Figure 7). All AchiIRs were on the same branch as HhalIRs. Thus, AchiIRs (5–12) were on the same branch as HhalIRs (2bA, 2bB, 2e, 2eA, 2eB, and 2eC), AchiIR1 was on the same branch as HhalIRs (2bD, 2d, 2dA, and 2dB), AchiIRs (2 and 3) were on the same branch as HhalIRs (1a and 2a), and AchiIR4 was on the same branch as HhalIR2aA (Figure 8). Similarly, all AchiGRs were on the same branch as HhalGRs, for example, AchiGR1 and HhalGR63b and AchiGR2 and HhalGR68c (Figure 9).

### Identification of Candidate SNMPs

**FIGURE 10:**
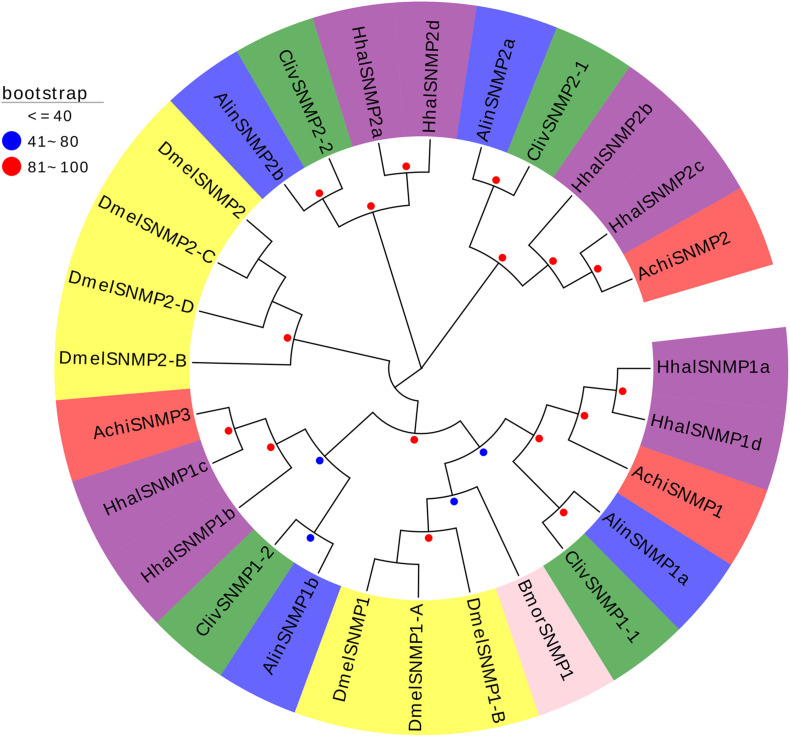
Phylogenetic analysis of the SNMPs (sensory neuron membrane proteins) from six insect species: *A. chinensis* (Achi, red), *D. melanogaster* (Dmel, yellow), *H. halys* (Hhal, purple), *C. lividipennis* (Cliv, green), *A. lineolatus* (Alin, blue), and *B. mori* (Bmor, beige). Red circles mean bootstrap values are 81–100, blue circles mean bootstrap values are 41–80, and hide bootstrap values are less than 41.

Three SNMPs were identified from the dataset of the *A. chinensis* antennal transcriptome. The protein sequence and BLASTX results are presented in Supplementary Tables 4, 5. The AchiSNMPs showed very high (89–94%) sequence identity with HhalSNMPs (Supplementary Table 5). A phylogenetic tree was constructed to better understand the relationships of the AchiSNMP proteins with the corresponding proteins of other five species: one Pentatomidae (*H. halys*), two Miridae (*C. lividipennis*, *A. lineolatus*), one Drosophilidae (*D. melanogaster*), and one Lepidoptera (*B. mori*) species. The results showed that the AchiSNMPs were located on three different branches, all AchiSNMPs were in the same branch with AlinSNMPs, ClivSNMPs, and HhalSNMP, and AchiSNMPs were closer to HhalSNMPs than to AlinSNMPs and ClivSNMPs. Meanwhile, AchiSNMP1 was in the same branch with BmorSNMP1 and DmelSNMPs (1, 1-A, and 1-B) (Figure 10).

### Sex-Specific Expression of *A. chinensis* Chemoreception Genes

**FIGURE 11:**
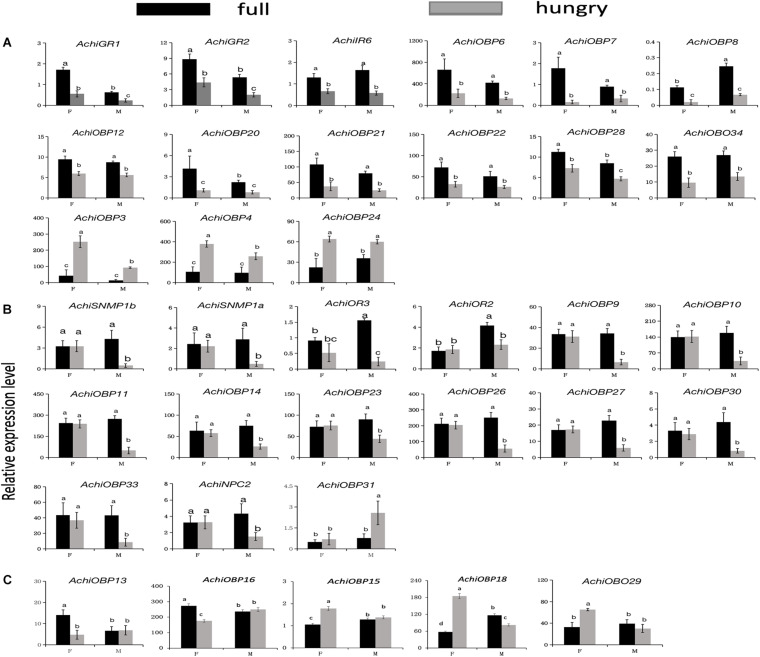
Sex-specific relative expression levels of *A. chinensis* chemoreception genes. Olfactory genes expressed in both males and females **(A)**, predominantly in males **(B)**, and predominantly in females **(C)**. Data are presented as the mean of three replicates (*n* = 3) ± SE. Different lowercase letters indicate significant differences among the four groups (*P* < 0.05). F, female antennae; M, male antennae. Black bar, full *A. chinensis*; gray bar, hungry *A. chinensis*.

The qRT-PCR assay results showed the significantly lower expression of *AchiGR1*, *AchiGR2*, *AchiIR6*, and *AchiOBPs* (*6–8*, *12*, *20–22*, *28*, and *34*) in the antennae of hungry *A. chinensis* and the opposite result for *AchiOBPs* (*3*, *4*, and *24*) (Figure 11A). The expression of *AchiSNMPs* (*1a* and *1b*), *AchiORs* (*2* and *3*), *AchiOBPs* (*9–11*, *14*, *23*, *26*, *27*, *30*, and *33*), and *AchiNPC2* in the antennae of full males and *AchiOBP13* and *AchiOBP16* in the antennae of full females was significantly higher than that in the antennae of their hungry male and female counterparts (Figures 11B,C). However, the expression of *AchiOBPs31* in the antennae of full males and *AchiOBPs* (*15*, *18*, and *29*) in the antennae of full females was significantly lower than that in the antennae of hungry males and females, respectively (Figures 11B,C). The expression of *AchiGR1*, *AchiGR2*, and *AchiOBP28* was significantly lower in males than in females whether full or hungry (Figure 11A).

## Discussion

In this study, 60 olfactory genes (38 OBPs, 1 CSP, 1 NPC2, 3 ORs, 12 IRs, 2 GRs, and 3 SNMPs) were identified in a transcriptome analysis of the antennae of male and female *A. chinensis* under full and hungry conditions. The sequences were compared with those of the corresponding genes in other hemipteran insects. *A. chinensis* contained fewer OBPs than *Apolygus lucorum*, but more than *Yemma signatus*, *C. lividipennis*, *Nysius ericae*, *H. halys*, *Corythucha ciliata*, *Acyrthosiphon pisum*, *A. lineolatus*, *Sitobion avenae*, *N. lugens*, and *Riptortus pedestris* (Supplementary Table 6) (Wang et al., 2010; Gu et al., 2011; Xue et al., 2014; Yuan et al., 2015; Paula et al., 2016; Xiao et al., 2016; Zhang et al., 2016; Song et al., 2017; Yang et al., 2018; Song and Sun, 2019). Only one CSP gene was identified in *A. chinensis*, unlike in *N. lugens*, *Y. signatus*, *C. lividipennis*, *N. ericae*, *C. ciliata*, *R. pedestris*, *A. pisum*, and *Sogatella furcifera* (Supplementary Table 6) (Wang et al., 2010; Gu et al., 2011; Xue et al., 2014; Yuan et al., 2015; Paula et al., 2016; Xiao et al., 2016; Zhang et al., 2016; Song et al., 2017; Yang et al., 2018; Song and Sun, 2019). Fewer chemosensory receptor genes were identified in *A. chinensis* (3 ORs, 12 IRs, and 2 GRs) than in other hemipteran insects, such as *C. lividipennis*, *R. pedestris*, *N. ericae*, and *C. ciliata* (Supplementary Table 6) (Wang et al., 2010; Gu et al., 2011; Xue et al., 2014; Yuan et al., 2015; Paula et al., 2016; Xiao et al., 2016; Zhang et al., 2016; Song et al., 2017; Yang et al., 2018; Song and Sun, 2019). The number of SNMP genes in *A. chinensis* (3) was less than that in *R. pedestris* and *A. lineolatus*, the same as that in *Y. signatus*, and more than that in *N. ericae* (Supplementary Table 6) (Wang et al., 2010; Gu et al., 2011; Xue et al., 2014; Yuan et al., 2015; Paula et al., 2016; Xiao et al., 2016; Zhang et al., 2016; Song et al., 2017; Yang et al., 2018; Song and Sun, 2019). This may be related to different species or to different methods, but differences in the depth of next-generation sequencing cannot be ruled out (Ge et al., 2016), because the transcriptome datasets cannot represent non-expressed or low-level-expressed chemosensory genes nor low transcript abundances (Li et al., 2015; Zhao et al., 2016). The specific functions of the identified OBPs, CSPs, NPC, ORs, IRs, GRs, and SNMPs in *A. chinensis* remain to be determined.

Our results also showed that the chemosensory genes of *A. chinensis* were largely distinct from those of other insects, such as *C. lividipennis* and *A. lineolatus*, which may reflect differences in prey preferences (Zhou et al., 2015). *A. chinensis* preferably preys on coleopteran, hemipteran, hymenopteran, and lepidopteran insects (Gao et al., 2011; Zou et al., 2012), *C. lividipennis* on the eggs of the green leafhopper and brown planthopper in rice (Heong et al., 1990), and *A. lineolatus* feeds on plants (Zhang et al., 2015). However, according to the NR database, the sequences of the antennal unigenes of *A. chinensis* matched well with those from *H. halys* (83.52%), and the phylogenetic trees revealed similarities between the chemosensory genes of *A. chinensis* and those of *H. halys* and *N. viridula*, suggesting a close ancestor of all three species. Moreover, comparisons of the appearances of *H. halys* and *A. chinensis*, together with the findings of previous studies, revealed the physical similarities of the two species (Zou et al., 2013). Accordingly, *H. halys* and *A. chinensis* may have had a common original ancestor who was either sarcophagus or phytophagous and during evolution became sarcophagus *A. chinensis* or phytophagous *H. halys*. The evolutionary relationship of *A. chinensis* and *H. halys* merits further research to obtain a better understanding of the evolutionary relationship of sarcophagy and phytophagy in insects.

Previous studies have demonstrated sex-based differences in the chemosensory genes of insects, such as *Tessaratoma papillosa* (Wu et al., 2017), *C. lividipennis* (Wang et al., 2018), and *A. lucorum* (Ji et al., 2013). In insects, the perception of chemical cues, including sex pheromones and plant volatiles, is important to find a conspecific partner and food sources, respectively (Yang et al., 2016; Chang et al., 2017; Wang et al., 2018). In our study, sexual differences were determined in 29 chemosensory genes of *A. chinensis*, with *AchiGR1*, *AchiGR2*, and *AchiOBP28* expressed at higher levels in the antennae of females than males regardless of whether the insects were full or hungry. The higher-level expression of *AchiOBP13* and *AchiOBP16* only in full female *A. chinensis* suggests that these genes encode proteins related to oviposition selection behavior (Pelosi et al., 2006; Wu et al., 2017; Wang et al., 2018). *AchiGRs* (*1* and *2*), *AchiIR6*, and *AchiOBPs* (*6–8*, *12*, *20–22*, *28*, and *34*) were predominantly expressed in full male *A. chinensis* and may thus be involved in mate searching (Liu et al., 2012; Gu et al., 2014; Wang et al., 2018) or the recognition of pheromone molecules (Liu et al., 2015). The significantly higher antennal expression of *AchiOBP31* in hungry males, *AchiOBPs* (*15*, *18*, and *29*) in hungry females, and *AchiOBPs* (*3*, *4*, and *24*) in hungry males and females indicates that these genes are involved in the search for food (Gu et al., 2014; Xue et al., 2016). Several genes, such as *AchiCSP1*, *AchiGR3*, and *AchiOBPs* (*1*, *2*, and *5*), did not differ significantly in their expression and may thus encode basic functions related to the binding of general-purpose volatiles (Gu et al., 2014; Xue et al., 2016). Further research is required to confirm the function of the sex-specific genes of *A. chinensis*.

*Arma chinensis* is an excellent predatory insect (Rider and Zheng, 2002) widely applied in China in the control of agricultural and forest pests (Gao et al., 2011; Zou et al., 2012). Improving its pest-control efficiency and its colonization of the target area are current areas of research and practical interest. Insects release sex pheromones to attract the opposite sex (Yatsynin et al., 2010; Kakizaki and Sugie, 2013), and different insects release different sex pheromones. The main sex pheromones of female click beetles are 8-hydroxygeraniol, 8-hydroxynerol (E,E)-farnesol, and all-trans geranylgeraniol (Yatsynin et al., 2010), whereas those of *Heliothis maritima adaucta* Butler are (Z)-11-hexadecenal (Z11-16:Ald), (Z)-11-hexadecen-1-ol (Z11-16:OH), and n-hexadecanal (n-16:Ald) (Kakizaki and Sugie, 2013). The application of insect sex pheromones in the target area may increase colonization, but the main sex pheromones of *A. chinensis* have yet to be identified. Our study found significant difference in the expression of the olfactory genes of full and hungry *A. chinensis*, but further research is needed to elucidate the composition of *A. chinensis* sex pheromones and their relation to olfactory genes. The results would allow the laboratory synthesis of *A. chinensis* sex pheromones and their application to improve the efficiency of *A. chinensis* in pest control.

## Conclusion

In summary, using next-generation sequencing technology, we identified 38 OBP, 1 CSP, 1 NPC2, 3 OR, 12 IR, 2 GR, and 3 SNMP genes from *A. chinensis*, an important insect predator used in forest and agricultural management. The data provide the basis for functional studies, whereas the phylogenetic trees created from the sequencing results will lead to new insights into the differentiation and evolution of the chemosensory systems of Hemiptera insects.

## Data Availability Statement

The datasets generated for this study can be found in the NCBI BioProject PRJNA668276, Biosample IDs: SAMN16393555–SAMN16393566.

## Ethics Statement

All applicable international, national, and/or institutional guidelines for the care and use of animals were followed.

## Author Contributions

SW, WD, ML, ZZ, and YL performed the experiments. SW, YX, JL, KT, ZX, and XL conceived and designed the experiments. SW, WD, YL, and ZZ analyzed the data and wrote the manuscript. All authors read and approved the final manuscript.

## Conflict of Interest

SW, YX, JL, KT, ZX, and ZZ were employed by Hunan Province Tobacco Company. The remaining authors declare that the research was conducted in the absence of any commercial or financial relationships that could be construed as a potential conflict of interest.

## Footnotes

1 www.ncbi.nlm.nih.gov/tools/primer-blast/
