# Supplementary material for: Analysis of Chemosensory Genes in Full and Hungry Adults of Arma chinensis (Pentatomidae) Through Antennal Transcriptome
Source: Front Physiol. 2020 Nov 6;11:588291. doi: 10.3389/fphys.2020.588291 (PMC7677363; doi:10.3389/fphys.2020.588291)
Supplement: Supplementary file 1 [file Table_1.DOCX]

Supplementary Table S1. The species used to constructed the phylogenetic tree of different Chemosensory Genes

| Chemosensory Genes | species |
| --- | --- |
| *OBPs* | *Arma chinensis, Nezara viridula, Halyomorpha halys, Cyrtorhinus lividipennis, Adelphocoris lineolatus, Drosophila melanogaster* and *Bombyx mori* |
| *CSPs* | *Arma chinensis*, *D. melanogaster*, *C. lividipennis*, *Adelphocoris lineolatus*, *N. viridula*, *B. mori* |
| *NPC2* | *Arma chinensis*, *D. melanogaster*, *H. halys*, *C. lividipennis*, *Adelphocoris lineolatus*, *Macrocentrus cingulum*, *Microplitis mediator*, *Camponotus japonicus*, *Operophtera brumata* |
| *ORs* | *Arma chinensis*, *D. melanogaster*, *H. halys*, *Cyrtorhinus lividipennis*, *Adelphocoris lineolatus*, *B. mori* |
| *GRs* | *Arma chinensis*, *D. melanogaster*, *H. halys*, *C. lividipennis*, *Adelphocoris lineolatus*, *B. mori* |
| *IRs* | *Arma chinensis*, *D. melanogaster*, *H. halys*, *C. lividipennis*, *Adelphocoris lineolatus*, *B. mori* |
| *SNMPs* | *Arma chinensis*, *D. melanogaster*, *H. halys*, *C. lividipennis*, *Adelphocoris lineolatus*, *B. mori* |
