# Supplementary material for: Analysis of Chemosensory Genes in Full and Hungry Adults of Arma chinensis (Pentatomidae) Through Antennal Transcriptome
Source: Front Physiol. 2020 Nov 6;11:588291. doi: 10.3389/fphys.2020.588291 (PMC7677363; doi:10.3389/fphys.2020.588291)
Supplement: Supplementary file 2 [file Table_2.DOCX]

Supplementary Table S2. Summary of the transcriptome sequencing data from Arma chinensis samples

| **Sample** | **Total Raw Reads (M)** | **Total Clean Reads (M)** | **Total Clean Bases(Gb)** | **Clean Reads Q20(%)** | **Clean Reads Q30(%)** | **Clean Reads Ratio(%)** |
| --- | --- | --- | --- | --- | --- | --- |
| Full female-1 | 49.19 | 44.37 | 6.66 | 96.3 | 87.16 | 90.21 |
| Full female-2 | 49.19 | 44.21 | 6.63 | 96.2 | 86.77 | 89.88 |
| Full female-3 | 49.19 | 44.13 | 6.62 | 96.17 | 86.78 | 89.71 |
| Hungry female-1 | 49.19 | 44.18 | 6.63 | 96.34 | 87.13 | 89.82 |
| Hungry female-2 | 47.43 | 42.87 | 6.43 | 96.32 | 87 | 90.39 |
| Hungry female-3 | 49.19 | 44.21 | 6.63 | 96.32 | 87.06 | 89.87 |
| Full male-1 | 49.19 | 43.64 | 6.55 | 96.25 | 87.02 | 88.72 |
| Full fale-2 | 47.18 | 42.95 | 6.44 | 96.21 | 86.81 | 91.03 |
| Full fale-3 | 47.43 | 43.11 | 6.47 | 96.98 | 88.64 | 90.88 |
| Hungry fale-1 | 49.19 | 44.89 | 6.73 | 96.88 | 88.27 | 91.27 |
| Hungry fale-2 | 47.43 | 43.05 | 6.46 | 97.46 | 89.98 | 90.76 |
| Hungry fale-3 | 47.43 | 42.88 | 6.43 | 97.53 | 90.15 | 90.4 |
