# Supplementary material for: Analysis of Chemosensory Genes in Full and Hungry Adults of Arma chinensis (Pentatomidae) Through Antennal Transcriptome
Source: Front Physiol. 2020 Nov 6;11:588291. doi: 10.3389/fphys.2020.588291 (PMC7677363; doi:10.3389/fphys.2020.588291)
Supplement: Supplementary file 3 [file Table_3.DOCX]

Supplementary Table S3. Summary of unigenes from the transcriptome sequencing data of *Arma chinensis*

| Sample | Total Number | Total Length | Mean Length | N50 | N70 | N90 | GC(%) |
| --- | --- | --- | --- | --- | --- | --- | --- |
| Full female-1 | 26277 | 24428077 | 929 | 1504 | 914 | 376 | 35 |
| Full female-2 | 23457 | 19776624 | 843 | 1346 | 784 | 342 | 35.37 |
| Full female-3 | 23052 | 18798088 | 815 | 1284 | 741 | 333 | 35.66 |
| Hungry female-1 | 25982 | 22596459 | 869 | 1380 | 820 | 355 | 35.11 |
| Hungry female-2 | 20743 | 15675827 | 755 | 1151 | 665 | 315 | 35.65 |
| Hungry female-3 | 25665 | 22029422 | 858 | 1367 | 808 | 350 | 35.31 |
| Full male-1 | 25253 | 21909517 | 867 | 1398 | 816 | 351 | 35.89 |
| Full fale-2 | 22691 | 19416097 | 855 | 1353 | 795 | 352 | 35.72 |
| Full fale-3 | 23992 | 20212479 | 842 | 1347 | 782 | 343 | 35.72 |
| Hungry fale-1 | 24339 | 21147097 | 868 | 1406 | 819 | 352 | 35.39 |
| Hungry fale-2 | 21621 | 17213992 | 796 | 1238 | 720 | 329 | 35.46 |
| Hungry fale-3 | 19889 | 14438513 | 725 | 1085 | 630 | 305 | 35.78 |
| All-Unigene | 34407 | 40468637 | 1176 | 1932 | 1224 | 510 | 35.14 |
