# Supplementary material for: Analysis of Chemosensory Genes in Full and Hungry Adults of Arma chinensis (Pentatomidae) Through Antennal Transcriptome
Source: Front Physiol. 2020 Nov 6;11:588291. doi: 10.3389/fphys.2020.588291 (PMC7677363; doi:10.3389/fphys.2020.588291)
Supplement: Supplementary file 4 [file Table_4.DOCX]

Supplementary Table S4. The protein sequence of AchiOBPs, AchiCSPs, AchiNPC2, AchiORs, AchiIRs, AchiGRs and AchiSNMPs

| Genes | Protein sequence |
| --- | --- |
| AchiOBP1 | MKTMTSLSLFLALFIAAKCDITSEATTTPQPSGGSTPAGGVTVSKSPEEIRQKIKEQVLALTESCKTSTKITAEQAKIVSNQAIPKTEAEKCFLECMYNGLNITRDGKFIELSAKGLAQHRFANSPDELTKANNMIETCTKEAVVKDANEKCAIGRLVRECFVKNGSKINFFPKP* |
| AchiOBP2 | MKTMTSLSLFLALFIAAKCDITSEATTTPQPSGGSTPAGGVTVSKSPEEIRQKIKEQVLALTESCKTSTKITAEQAKIVSNQAIPKTEAEKCFLECMYNGLNITRDGKFIELSAKGLAQHRFANSPDELTKANNMIETCTKEAVVKDANEKCAIGRLVRECFVKNGSKVIILIHVVYDT* |
| AchiOBP3 | LCKIMSRSLLCFVTLLITAHMALSYFPDEWETDCKKENGYEGDIGDLNYTDPSSVPRPAMCYMACFMGKQKVMKPDGGIDFEYAKKLYSTVHKGDEKIIRRFHWMVDQCAKEFKQYPDKCETAFYYVKCKREKFDAYKE* |
| AchiOBP4 | LCKIMSRSLLCFVTLLITAHMVLSYFPDEWETDCKKENGYEGDIGDLNYTDPSSVPRPAMCYMACFMGKQKVMKPDGGIDFEYAKKLYSTVHKGDEKIIRRFHWMVDQCAKEFKQYPDKCETAFYYVKCKREKFDAYKE* |
| AchiOBP5 | KQFSSLFCAVALISVLGLQLVLALTEDECTLTPDQVPVCCKEPKPTEEIKEDKAREELGKKCVENNTKGKKANTEAEEFQIAECVDECIFRDVFGYVNKKTNKLDETAIVNTFTKRFDGNAKWKEATQKVAKNCLGESGKDVKASSKCKSGALQFLRCYTRGVFLNCPAESWENSELITMLIADVTIIVFIIIFLFILQMMPVTSSKFSYRSVQMSIPFCKTKVSC* |
| AchiOBP6 | MKQFSSLFCAVALISVLGLQLVLALTEDECTLTPDQVPVCCKEPKPTEEIKEDKAREELGKKCVENNTKGKKANTEAEEFQIAECVDECIFRDVFGYVNKKTNKLDETAIVNTFTKRFDGNAKWKEATQKVAKNCLGESGKDVKASSKCKSGALQFLRCYTRGVFLNCPAESWENNDACNKLKVLVQKCPNVYTLLQN* |
| AchiOBP7 | KQFSSLFCAVALISVLGLQAALLHLLVPVCRWTSSLPRVFSWIPIHKLPGPCYSIPSDDMSGPGPHPLMDFFHNIWKLVLALTEDECTLTPDQVPVCCKEPKPTEEIKEDKAREELG |
| AchiOBP8 | VFVAMKRAAILLIFFLTASSALADDEEVDMDVYDKILEDFDVDTIMQNDRLLDSYLKCFFNTGPCSEIAEMVKSKIPEVFSTVCGLCSPKQKELFKHCLTIFIPKRPDDWKHILEIYDPDGSYWPKIKEFLETY* |
| AchiOBP9 | VFVAMKRAAILLIFFLTASSALADDEEVDMDVYDKILEDFDVDTIMQNDRLLDSYLKCFFNTGPCSEIAEMVKSKIPEVFSTVCGLCSPKQKELFKHCLTIFIPKRPDDWKHILEIYDPDGSYWPKIKEFLDTN* |
| AchiOBP10 | TMFLAAVLTILLAVTLSPAYGEGEEEDLYKKIFDDVDIDSILNNDRILDTYLRCFFNVGPCSNLAETMRSKIPEVFSTVCGLCTDKQKGLFKHSLDIFIPKRPDDWKHILEIYDPDGSYWPKIKEFLETY* |
| AchiOBP11 | LHVFYLLAAFAFASGVDIPDELKEMGQMVHDQCIGETGASNDAIEATKKGVFPADDQKLKCYLKCIYSNMGAISDDGELDAEAFSSVMPEELGAVLNPMIEKCKGVTGADGCELAFNFNICLYNADPKNYLVI* |
| AchiOBP12 | ATMARFFLLLLATCFLGAFGVSFSGNKIEFKNATWGKCQKEWNAPDEAMRDFYIFRSMEGLKNYKCVVKCINEEYGVYLKNGTVSKPKLLHIIKYLTKDPKTLEKLLSTTEECVDSVLQIPDQCQLNYDLVDCYLVNMKKNEIHLG* |
| AchiOBP13 | KMAPMAQWSLLVLATFILGAFAAPGTDNPHASKNATWAKCQKEENAPADAMRDLYIFKSTEGLKDHKCVAKCINEEYGIFVKGTIRKEVIDKIIKYLWKDAQAQEKLINISDSCQDSVNLELDPCQINYDMFSCFIRGFQKDGFVLV* |
| AchiOBP14 | KMTPMAQWSLLVLATFILGAFAAPVIDDPVAFKNATWEKCQKEENAPADAMKDLYIFKSAEGIKDHKCVAKCINEDYGIFEKDGTISKEVIDKTIKGLWKDAQAQEKLISISDACVDSVNRELDPCEINYDLIECFILGIQKDGIVVV* |
| AchiOBP15 | MYTTNLIFLVVLVASAVGWPQPPPPAGAEDVPEECRPKPPQHGKERTVCCDMPNPISMNTDKFKTVFEECKEQIKSQRPDVMPPNHSHPPPPPPSAGGPSPPHRGPHHGPPRFIIECMEECVFNKSGLITDSKLNEEALTKLVDTYVENSAWKPIALEATKHCYEKRDETTAEDGKCNPGAHEFTKCMIKQMYLVS* |
| AchiOBP16 | LAMYTTNLIFLVVLVASAVGWPQPPPPPGAEDVPEECRPKPPQHGKERTVCCDMPNPISMNTDKFKTVFEECKEQIKSQRPDVMPPNHSHPPPPPPSTGGPSPPHRGPHHGPPRFIIEVCIL* |
| AchiOBP17 | LAMYTTNLIFLVVLVASAVGWPQPPPPPGAEDVPEECRPKPPQHGKERTVCCDMPNPISMNTDKFKTVFEECKEQIKSQRPDVMPPNHSHPPPPPPSTGGPSPPHRGPHHGPPRFIIEVCIL* |
| AchiOBP18 | RRVPSSEASLAMYTTNLIFLVVLVASAVGWPQPPPPAGAEDVPEECRPKPPQHGKERTVCCDMPNPISMNTDKFKTVFEECKEQIKSQRPDVMPPNHSHPPPPPPSTGGPSPPHRGPHHGPPRFIIECMEECVFNKSGLITDSKLNEEALTKLVDTYVENSAWKPIALEATKHCYEKRDETTAEDGKCNPGAHEFTKCMIKQMYLVS* |
| AchiOBP19 | RRVPSSEASLAMYTTNLIFLVVLVASAVGWPQPPPPAGAEDVPEECRPKPPQHGKERTVCCDMPNPISMNTDKFKTVFEECKEQIKSQRPDVMPPNHSHPPPPPSAGGPSPPHRGPHHGPPRFIIECMEECVFNKSGLITDSKLNEEALTKLVDTYVENSAWKPIALEATKHCYEKRDETTAEDGKCNPGAHEFTKCMIKQMYLNCPSDKWQDSKYYCILNRRKKFDI* |
| AchiOBP20 | MNPALALTTLLAVICLAGAAAPEYKAKVIAAITTCAKEYNVDLKEVCEFAKLNKLPETKEQKCVVGCTYEKMGYVADGKIDWEKVKALNSQKYDTPELVEKVNQVTDACSKVVTEKSDDVCDLGLQAIKCMMEESEKVQLPKPDIKIESE* |
| AchiOBP21 | MNPTLSLTTLLAALCLAGAATPEYKAKVITAVTTCSKEHNADLKDVIEIMRQNKLPETKEHKCVVGCFYEKMGYVTDNKVDWEKVKALNSQKYDTPELVEKVNQVTETCSKVVTEKSSDICELGIPAIKCLKEEAEKVQLPKPDIKFDSA* |
| AchiOBP22 | KMNPALSLTTLLAALCLAGAATPEYKAKVVTAVTTCAKENNVELKEVIEIMKQSKLPESKEQKCVVGCFYEKMGYMTDKKIDWVKVKALNSQKYDTPELVEKVNQITDTCSKVVTEKSDDICDLGVQAIKCLKEEADKVQLPKPDIKFDSA* |
| AchiOBP23 | MNPALSLTTLLAALCLAGAATPEYKAKVVTAVTTCAKENNVELKEVIEIMKQSKLPESKEQKCVVGCFYEKMGYMTDKKIDWVKIKALNSQKYDTPELVEKVNQITDTCSKVVTEKSDDICDLGVQAIKCLKEEADKVQLPKPDIKFDSA* |
| AchiOBP24 | MRRSLLCFVTFLVTVHVALSFFPDKWVEDCKKENGYVGDIGELNYTDPSSVPRAAKCYMACFMGKQHVMKPDGSIDFEYAKLLYSIVYKGDEKKISMFHRIVDECAKEYKQYTDKCETALHYVKCKRLKYAANNN* |
| AchiOBP25 | GKMKCIITALALALIFTTTEAEDSVKEKIMKVFNSCKEKHPITDDELGAFRKADVGYGYSHDAKCMLACMLEEGKILIDGRYMKQNALIMADVFHMDDFDEAAKARKVIEVCAEQVPEVGSDQCEFAYKMASCGANESKKVSDVNM* |
| AchiOBP26 | GKMKCIITALALALIFTTTEAEDSVKEKIMKVFNSCKEKHPITDDELGAFRKADVGYGYSHDAKCMLACMLEEGKILIDGRYMKQNALIMADVFHMDDFDEAAKARKVIEVCAEQVPEVGSDQCEFAYKMASCGANESKKLGMKDHDFFE* |
| AchiOBP27 | RTSDKMKSFLVIVALSAFIVLAKGDDIKEKFKKTAEKCKEKHAISEDEIQKLKMKDTEYQYSHVAKCYMACFLEEGKILQNGKYNKENALVMADVIHKENPDEAAKAKEIIETCAKQYPDVGADQCEFAYNVSVCAAKEAKKMGLDNTEFYTK* |
| AchiOBP28 | AMQLVILASVLALAAAAKTQAKQQNCVAPTTAPHKIEKVLSQCQDEIKYALLQEAVSVLGQTVGRQKRETFTGEERRIAGCLLQCVYRKMKALDENGFPTAPALVEMFTEGVKDRNYYLATIQGVQQCLAKEIQQRKSNQTLAEAEGYTCDVAYDMFMCVSEQIEILCGISP* |
| AchiOBP29 | PLSALLVTICVFAVVRAKDPVDCSKPPPGWPRQPPNCCDQPYPTDQMRKHLVNCIRQYGAPSSAVLTEKSIRERRSCVEECVYRAAGFINKEDSVLQREALEEQLKSVAGQSWEKAITESLDACFKEAEEFEDSLSASSSEEEESSCSSTPERLTFCLSRQLFLNCPEDTWKNTQECQVVKNRMQECKQLLPPPPVRFIRPGPRPPIGGQ* |
| AchiOBP30 | ELTIFNYMLAMVLAVAVVDGSVDGPEAELSDKELMGLAGMHYYSKLSSRNARDMDDHHHHKKHLSYKHCCGGAENRNHTGKKETWKIVKKCIEQEKEKFNDTEQFVNPITNEFSCERLKRDKNRHYCVADCVMKEVGALQGDDAVNKDIAKEFLTKGISAEWLKEIAEKGVERCAEQKDVTIFNGDQLECNPWAGYIRHCMWKEVILHCPEEHFNNSAYCTRVKKSFEEMNSVISKK* |
| AchiOBP31 | MTPMAQWSLLVLATFIVGAFAAPVIEDPLAFKNATWEKCQKQQNVPDGAMNDLYLFKSVEGIKDYKCAAMCLEEEYGAYQKGGQIKKEVIQDAIRGLWKDTQTQSKLITIADDCVDAANYDLDPCEMNYNLFDCFIESMQKDGLQVEKIS* |
| AchiOBP32 | EMAAMAQWYLLVFATLVFGASAIPLTEDAIAFRNATFKRCAIEENAPADAMVELYYFRSAEGVKDHKCVTKCIDEAFGIYEKDGTFNKEVIAVAIESLWDEPKVKQELRIASGACVDSLNYSLDPCEVNYDLFDCLIKIIKEEGIEVF* |
| AchiOBP33 | MKVYIINFLIVISMSFSFSSTVKPGIISQAFQIVGRCNKEHPLDMKEMEKAVKNFELPTSEEGKCFISCFLEHFGLITDRQVNLEKSMEFNKMQFHNPDDLEKANTISATCKDELSTSTEEGCDFAIAASKCMLEKSKEMGFRFFQFHS* |
| AchiOBP34 | MNSLLFLTALLSAVCLSWAASDEYKLKVIQSVAACVKEHGANLVEVIQVVHADKLPTNKAQKCVAGCFFNKMGYVTDSKVDWAKVKALNPKKYDTKELVAKADKVVDACAKAVTEKKTDVCELGLAPMKCLLEQSKKVQLPKPEI |
| AchiOBP35 | KMTPMAQWSLLVLAIFILGACAAPVIDDPLAFMNATWEKCQKEENAPPDAMKELYTFTSAKGIKDHKCVTKCINEEYGIFDKDGTISKEVIDKTIKGLWKDTQAQEKLTSMSDACIHSVNLELDPCQINYDMFSCFIRGFRSFR* |
| AchiOBP36 | RFGNTANMKYLVLSLVLCYAYAGSIEESKNLLKKELQVFGKCMADGNVTESVVENILKKGEVPETRQVKCLLACHMKGMGYLSNDGKMDWSKLDDINKIEYVDPEHVKKALEVDAVCKKKVPENLGNPCEVAFAVTKCFIEEAKKRNVPILGPESAQ* |
| AchiOBP37 | MEKSLLLFSVLLMACTLVQESRAAMSEAQMKNAMKTLRNMCMPKSGVSKEALASMKEGQFDDDDRKLKCYLGCIMNMMQVVKNGKISMTMVKNQITKMVDPAWGAKLVSTFESCANVEGSDNCDLAYNFGKCVYETDKDAFVVP* |
| AchiOBP38 | KARQLLFKIMTAILVLAFAFLCTGLVSAGVKEELGHQLEECKSSFNVTDDEIKGITLKQPPTSQAGKCYLHCIFSRMDVMTEEGNMNTEGMKGVIREIPEIKESDIQKMEEVADKCAEAPLGEDKCENAVTIFNCINSEADKLGVKGSP* |
| AchiCSP1 | TSWAIGVVLLVALESCKGDLLTQGQKQRINSADVKRMLTDRNYVLKQINCVLHDVECDEIGLHLKRVIPDVLIKNCASCSPQEAQTARNIINYIRGKYPNEWAEIQS |
| AchiNPC2 | KAQIALNNGSEGMKAWENLPLPLEFKVFIFNITNPDEVSKGMKPVVQELGPYVYDQYRRKVDIEFNEDDTISYRIEKKFYFNRNKSGCHRESDVIVVPNLPLIGTAYGIEERFPMGLVFINSTAHLLFPGIKNLFLTTTVGDLLFNGVRIKCDYMKGPAMPVCQGIKRNLPPSLKEIPSSGDFAFSYFSDANSSVTGVFKAYRGNENVYDLGRIVEYNNHSDLIMWDKNTTCSELRGTDSTILPPIQNKDQDIYIFLPELCLSLKAVFSKETTMYGIDVYHYIASHHNFDSEKTNPSNICRCKKQEDDPTSPPTCLKDGAIDASRCQGAPVVFTYPHMLYADPEYQNFVEGYHSDYGKHQTEVFIEPRTGVPLAAFKRVQMNIFLRRLNDVDLFANISEGLIPLIWIEEALTEELVQTYLPDMKEMMSITRIIMSVTGLLIGIGIFCLLVALILYLKHRKVECIQENQVVSNISLIGTGKATRNDPDAMGKRISYDLPNTVGNDRVTVQKL* |
| AchiOR1 | EHQRVERSVIQCLRNTGISLPWLDGDSVTWRVLRNFYNYLVFLIVVYHLIIAIMSAILLPDFDGKCRAGALISVTSNPTVISIYYALYHKRIQKFFEHSDNLNQQILDSELGEKDYFETIYLKSAKKINVLTVSIVMFIFMTPIIYNIPRPIIEIYNHEYRKTQPVYLYYPFDVHLPGSYEVTFLLQTLSLLCGDIKKFAND |
| AchiOR2 | EHQRVERSVIQCLRNTGISLPWLDGDSLTWRVLRNFYNYLVFLIVVYHLIIAIMSAVLLPDFDGKCRAGALISVTSNPTVISIYYALYHKRIQKFFEHSDNLNQQILDSELGETEYFKTIYLKSAKKINVLTVSIVMFIFMTPIIYNIPRPIIEIYYHEYRKTQPVYLYYPFDVHLPGSYEVTFLLQTLSLLCGDIKKFANDCFFLAMFRAQTVYLKYLSASIKDLGEQFKTSDNVVIRRKMINWINLHDQFIRNFNELMDLYTPVICIYKVNLICIVVLCIFTQLQDKKYGWIEGSGFAGFFLANIFQLYLQCETNDDFSVEAENLSLEIYKTPWYEIDKTNKDMLKTTLLMASRPVEITAFNSPMLMLNKETFLAFVGSTITAVMTIKEMSDLRQ* |
| AchiOR3 | LYIPGLGLLVGSSILMCMSGFIFVSKEVPFVSKFVFCSFLLSELLLIFLICWCGENIQNTSAQVFEMVYSSEWPSNIKSMDLYILIIQARTLNPIKISFGGIMVASLETYSNICSSAFSYFNILLAVN* |
| AchiIR1 | HLENQFTLLNCMWFAIGSLMQQGCDFLPKAVSTRMVAGMWWFFTLIMISSYTANLAAFLTVERMDSPIESAEDLAKQTKIKYGALRGGSTVAFFRDSNFSTYQRMWSFMESARPSVFTDSNSDGVERVTKGKGNYAFLMESTSIEYVIERTCDLTQIGGLLDSKGYGIAMPPNSPYRTAISSAVLKLQEEGKLHILKTKWWKEKRGGGACRDDALKSSSAANELGLANVGGVFVVLMGGMGVACVIAVCEFVWKSRKVAVEEREASFCTGMASEIRNAIHCKNNDPTKEALESKTPEKKDVPVFLPQGTYSQYGFLSNGPL* |
| AchiIR2 | MLDNPDVMMPNNDAGVDRVVSEEKYAFFMESASIEYEVQRKCQLAMVGDLLDSKGYGIVMRQNSTFRNALNKNVVRLQENGKLTQLKDKWWKEKRGGGACTSGTEEGEASELNLDNVGGVFVVLVAGCLIGVLLSFCEVLWDISQRDEKISFKHELIEEIKFIMKCKGTVKPVRKNSLTIANCNNLSKSSSKSSTTSKRTSKNSFSFRSPYS* |
| AchiIR3 | MLIKILLISFLLITKSYSSENEGNEEGIDNEEIVKTEVRIGALFDKDDETLIKAFNSAVELVNSDEELLPNMTLVPITFTGIPEYDSMEVGKKVCELMSYGVAAIFGPQSPFTSYHVQSLCDTMEMPHLSTKWDLSQRRSSCLLNIYPHPSTLTQAVTDIVTAWNWKGFTVLYDDFDALKKIQGIIKLADDKGYLVTVRQLEAEDGNYRAVLKEIKHSDETNIVIECAVEKLYDLLVQAQQVGIMGSHYSYIITTLDFQTINYEPFMWGGTNITGIRLVDPDDPYVINATQTEQRGEDGMESTTTNLDYIPTTEPPIEEEEEEGSSIPTVEAALLHDAVRLFVKALHHLSPLNIKPLTCGPHSSLDFGYTVINYMRLSEIKGLSGVIKFNHEGFRTDVQLDVISLTEEGLKKTGTWNTTTGLVMDPPDTNEGLVVDAGEDLRNKSFIVIIALTKPYNMLKEDSKTLTGNDRYEGFGVDLIHELSLMSGFNYTFVEQYDKNSGSPTTLQNGSRIWNGMIGEVQAGRADLAIADITITRERERDVDFTHPFMNLGISILHRKPSKAPPNLFSFLSPFSNDVWSCMLGAYFGVSLLLFVMARLSPYEWTNPYPCIEEPEHLENQFSLLNSLWFTLGSVMQQGSDVAPISVSTRMVASIWWFFTLIMVSSYTANLAAFLTVENNVSPFSDVKELAGQTEIEYGAKNNGATANFFRDSKEELYQKIYKFMLDNPDVMMPNNDAGVDRVVSEEKYAFFMESASIEYEVQRKCQLAMVGDLLDSKGYGIVMRQNSTFRNALNKNVVRLQENGKLTQLKDKWWKEKRGGGACTSGTEEGEASELNLDNVGGVFVVLVAGCLIGVLLSFCEVLWDISQRDEKISFKHELIEEIKFIMKCKGTVKPVRKNSLTIANCNNLSKSSSKSSTTSKRTSKNSFSFRSPYS* |
| AchiIR4 | EINSHNLEGKKDLHIMSDCVINMPISLGLQKNSPLKPSVDKFLQRVIEAGLIKKWLADVMLSTTVAEAPFEKNNINAVMDLKKFVGALVALGIGYGLGLIALIIENIYWYYVVQKNPLFDKYKQECNRKC* |
| AchiIR5 | ELTKNGFRKIGTWDPVKGISYTRTGSQMENEMFQSISNKTFYVVSRVGEPYLKEVDKNAEGNARYAGYSMDLIDEIAKDLKFSYKFYLAPDGEYGSFNKETKQWTGLIKELRERRADLGICDLTINYERRSAVDFTMPFMTLGISILYSKPMKQPPELFSFLSPFSVDVWVYMATAYLGVSLLLYFLARCTPDEWDNPHPCNPDPTELECIFSLHNCLWFSIGSLMAQGCDLLPKALSTRVVAGMWWFFVLIMISSYTANLAAFLTMDRMEATIESVEDLANQNKIKYGVLKKGSSANFFKDSNVSLYQKIWSQMESAHPSVFTNGNDEGVERVLKGNRGYAFFMESTTIEYQKEKHCTLMQVGGLLDSKGYGIAMPFNSPYRIAISGAVLKMQESGRLQQLKDKWWKNQKNKCPGEDKKKESSRLSIAHVGGVFLVLLVGCVVAFFVSILEFLWNVRKVAVTEKISPGEAFLLELKFAIQCYGTTKPVWNRREDSVVEKDFVENHEVEGEEEEEEDRGFFGDDNMEEEYMRMNGFNKNIRAMSTQSYS |
| AchiIR6 | VDKNAEGNARYAGYSMDLIDEIAKDLKFSYKFYLAPDGEYGSFNKETKQWTGLIKELRERRADLGICDLTINYERRSAVDFTMPFMTLGISILYSKPMKQPPELFSFLSPFSVDVWVYMATAYLGVSLLLYFLARISPMEWKNPHPCNKDPEELENTLAIYNAIWHNIGSLMQQGSDIAPQALSTRVVAGMWWFFVLIMISSYTANLAAFLTMDRMDATIESVEDLANQNKIKYGVLKGGSSANFFRDSNVSLYQKIWSQMESARPSVFTKSNDEGVERVLKGKRAYAFFMESTTIEYQKEKHCSLMQVGGLLDSKGYGIAMPFNSPYRIAISGSVLKMQESGRLLQLKDKWWKNSADKQNCPVEEAGTSSSELSIANVGGVFLVLLVGCVAAFFVAILEFLWNVRKVAVEEKISPSDAFFLELRFAIQCYGTTKPVRKPREESVAEEDIAECPEIEGAEEQEEEEEDRGFFGDENMEEQYMRMNGFSNKIRAKSTQSYS |
| AchiIR7 | IFSLHNCLWFSIGSLMAQGCDLLPKALSTRVVAGMWWFFVLIMISSYTANLAAFLTMDRMDATIESVEDLANQNKIKYGVLKGGSSANFFRDSNVSLYQKIWSQMESARPSVFTKSNDEGVERVLKGKRAYAFFMESTTIEYQKEKHCSLMQVGGLLDSKGYGIAMPFNSPYRIVMSCSVLKMQESGRLLQLKDKWWKNSADKQNCPVEEAGTSSSELSIANVGGVFLVLLVGCVAAFFVAILEFLWNVRKVAVEEKISPSDAFFLELRFAIQCYGTTKPVRKPREESVAEEDIAECPEIEGAEEQEEEEEDRGFFGDENMEEQYMRMNGFSNKIRAKSTQSYS |
| AchiIR8 | LTITYERRSAVDFTMPFMNLGVSILYSKPTKQPPDLFSFLLPFSIDVWTYMATAYLGVSLLLYFLARCTPDEWDNPHPCNPDPTELECIFSLHNCLWFSIGSLMAQGCDLLPKALSTRVVAGMWWFFVLIMISSYTANLAAFLTMDRMDATIESVEDLANQNKIKYGVLKGGSSANFFRDSNVSLYQKIWSQMESARPSVFTKSNDEGVERVLKGKRAYAFFMESTTIEYQKEKHCSLMQVGGLLDSKGYGIAMPFNSPYRIAISGSVLKMQESGRLLQLKDKWWKNSADKQNCPVEEAGTSSSELSIANVGGVFLVLLVGCVAAFFVAILEFLWNVRKVAVEEKISPSDAFFLELRFAIQCYGTTKPVRKPREESVAEEDIAECPEIEGAEEQEEEEEDRGFFGDENMEEQYMRMNGFSNKIRAKSTQSYS |
| AchiIR9 | ARISPMEWKNPHPCNKDPEELENTLAIYNAIWHNIGSLMQQGSDIAPQALSTRVVAGMWWFFVLIMISSYTANLAAFLTMDRMEATIESVEDLANQNKIKYGVLKGGSSANFFRDSNVSLYQKIWSQMESARPSVFTKSNDEGVERVLKGKRAYAFFMESTTIEYQKEKHCSLMQVGGLLDSKGYGIAMPFNSPYRIAISGSVLKMQESGRLLQLKDKWWKNSADKQNCPVEEAGTSSSELSIANVGGVFLVLLVGCVAAFFVAILEFLWNVRKVAVEEKISPSDAFFLELRFAIQCYGTTKPVRKPREESVAEEDIAECPEIEGAEEQEEEEEDRGFFGDENMEEQYMRMNGFSNKIRAKSTQSYS |
| AchiIR10 | VDKNAEGNARYAGYSMDLIDEIAKDLKFSYKFYLAPDGEYGSFNKETKQWTGLIKELRERRADLGICDLTINYERRSAVDFTMPFMTLGISILYSKPMKQPPELFSFLSPFSVDVWVYMATAYLGVSLLLYFLARISPMEWKNPHPCNKDPEELENTLAIYNAIWHNIGSLMQQGSDIAPQALSTRVVAGMWWFFVLIMISSYTANLAAFLTMDRMEATIESVEDLANQNKIKYGVLKKGSSANFFKDSNVSLYQKIWSQMESAHPSVFTNGNDEGVERVLKGNRGYAFFMESTTIEYQKEKHCTLMQVGGLLDSKGYGIAMPFNSPYRIAISGAVLKMQESGRLQQLKDKWWKNQKNKCPGEDKKKESSRLSIAHVGGVFLVLLVGCVVAFFVSILEFLWNVRKVAVTEKISPGEAFLLELKFAIQCYGTTKPVWNRREDSVVEKDFVENHEVEGEEEEEEDRGFFGDDNMEEEYMRMNGFNKNIRAMSTQSYS |
| AchiIR11 | VDKNAEGNARYAGYSMDLIDEIAKDLKFSYKFYLAPDGEYGSFNKETKQWTGLIKELRERRADLGICDLTINYERRSAVDFTMPFMNLGVSILYSKPTKQPPDLFSFLLPFSIDVWTYMATAYLGVSLLLYFLARISPMEWKNPHPCNKDPEELENTLAIYNAIWHNIGSLMQQGSDIAPQALSTRVVAGMWWFFVLIMISSYTANLAAFLTMDRMDATIESVEDLANQNKIKYGVLKGGSSANFFRDSNVSLYQKIWSQMESARPSVFTKSNDEGVERVLKGKRAYAFFMESTTIEYQKEKHCSLMQVGGLLDSKGYGIAMPFNSPYRIAISGSVLKMQESGRLLQLKDKWWKNSADKQNCPVEEAGTSSSELSIANVGGVFLVLLVGCVAAFFVAILEFLWNVRKVAVEEKISPSDAFFLELRFAIQCYGTTKPVRKPREESVAEEDIAECPEIEGAEEQEEEEEDRGFFGDENMEEQYMRMNGFSNKIRAKSTQSYS |
| AchiIR12 | WFSIGSLMAQGCDLLPKALSTRVVAGMWWFFVLIMISSYTANLAAFLTMDRMDATIESVEDLANQNKIKYGVLKKGSSANFFKDSNVSLYQKIWSQMESAHPSVFTNGNDEGVERVLKGNRGYAFFMESTTIEYQKEKHCSLMQVGGLLDSKGYGIAMPFNSPYRIAISGSVLKMQESGRLLQLKDKWWKNSADKQNCPVEEAGTSSSELSIANVGGVFLVLLVGCVAAFFVAILEFLWNVRKVAVEEKISPSDAFFLELRFAIQCYGTTKPVRKPREESVAEEDIAECPEIEGAEEQEEEEEDRGFFGDENMEEQYMRMNGFSNKIRAKSTQSYS |
| AchiGR1 | MEILAAIKHSDHNVQGEIKKIKVEMNERLTSIEKHLEGQNKYIEEILAENQALKARVGNLEVRLNKSEQGLLSKCLEIRGIPIRAGETPSGLVASIGAGLGLKLNIEDLDTVQRRRAKNDDPRPPPIIARFTRQSVRDDLIQK |
| AchiGR2 | MIQLLKAKDFSATLYQLMIDENTLFCQNKKIRLHLTVQKEPCFTAYGFFDLDFTLLHSIIAAATTYLVILIQFSQTTTSQKGILLNATAAYNLSNSNYTE |
| AchiSNMP1 | SDMAAPLRLGVAGAVLTFLGIIFGFWGFHKFLAYKINQNVALKKGNDMRAAWSKFPIPLEFRIYLFNVTNPQEVHAGQKPKLQEVGPYFFDEWKEKIKLEDDPAEDTVSFNQRAAWVFQESRSEGLTGEEVITIPHPALLSMVLTVEKQKPGALPMISKALPALFNSPSTVFLTAKVMDILFRGVPINCSSSDFGPKAICTMIRANPKGLKQLNDDIFLFSFFGVKNNTMEEGRFTVRRGIRDAKEVGSMVKFNGKEMQDVWSGPECNALRGTDSTIFPPFIDDSEDIVSFAPDLCRSLGAKFRHKIVYKGIPGNHYTADLGDMSSNPEEKCFCPTPTTCLKKGAFDISKCVGAPIVLTLPHFYETDQSYLDTVDGLHPEKEKHQIFLNFEPMTGTPLGARKRLQFNIPIHAIKKVALMKELPDALIPLFWVEEGLELDQKFIDILDAQLFRALRIVGIGRWVMVVLGLAMIGGGVGLHYYRKNKMIPPMVTQVSPPPNKY* |
| AchiSNMP2 | KAQIALNNGSEGMKAWENLPLPLEFKVFIFNITNPDEVSKGMKPVVQELGPYVYDQYRRKVDIEFNEDDTISYRIEKKFYFNRNKSGCHRESDVIVVPNLPLIGTAYGIEERFPMGLVFINSTAHLLFPGIKNLFLTTTVGDLLFNGVRIKCDYMKGPAMPVCQGIKRNLPPSLKEIPSSGDFAFSYFSDANSSVTGVFKAYRGNENVYDLGRIVEYNNHSDLIMWDKNTTCSELRGTDSTILPPIQNKDQDIYIFLPELCLSLKAVFSKETTMYGIDVYHYIASHHNFDSEKTNPSNICRCKKQEDDPTSPPTCLKDGAIDASRCQGAPVVFTYPHMLYADPEYQNFVEGYHSDYGKHQTEVFIEPRTGVPLAAFKRVQMNIFLRRLNDVDLFANISEGLIPLIWIEEALTEELVQTYLPDMKEMMSITRIIMSVTGLLIGIGIFCLLVALILYLKHRKVECIQENQVVSNISLIGTGKATRNDPDAMGKRISYDLPNTVGNDRVTVQKL* |
| AchiSNMP3 | YIYGKLGSVFMTAKVRELLFDGVLIDCTAKNIVPKAICIAIKQNSKALVKLGNNKYLFSFFGIRNATPEEARITVKKGVEDVYSIGKVVAMNGNPENVVWSGGECRRFSGTDSTIFPPFRKPDNYSIIAFSPEICRTMTGVYVGDGEYQGVRGYKYEVSLGDMKRNPGEMCFCPSPDRCLGKGTTDLTKCQGAPLIGSLPHFYDAEEDYLNGVEGMNPIKEKHEISFIMEPISGVPLLARKRLQFNIHLHPVRFINLTRKLTPTLVPIFWLEENLDLGDELMGFLEANLLTNLKLVDVVKWMLIVVGAGVCVAGVVLYRMKKEASKKADSKEDLLPPGSD |
