# Supplementary material for: Analysis of Chemosensory Genes in Full and Hungry Adults of Arma chinensis (Pentatomidae) Through Antennal Transcriptome
Source: Front Physiol. 2020 Nov 6;11:588291. doi: 10.3389/fphys.2020.588291 (PMC7677363; doi:10.3389/fphys.2020.588291)
Supplement: Supplementary file 5 [file Table_5.DOCX]

Supplementary Table S5. The BLASTX results of AchiOBPs, AchiCSPs, AchiNPC2, AchiORs, AchiIRs, AchiGRs and AchiSNMPs

| Gene names | Full length | Top Blastn hit | %ID | E-vale | score | TM |
| --- | --- | --- | --- | --- | --- | --- |
| OBP1 | YES | MK753154.1 Nezara viridula odorant binding protein 9 | 94.857 | 2.79E-93 | 282 | 3 |
| OBP2 | YES | MK753154.1 Nezara viridula odorant binding protein 9 | 94.083 | 4.93E-88 | 269 | 3 |
| OBP3 | YES | MK753161.1 Nezara viridula odorant binding protein 16 | 81.429 | 2.34E-79 | 243 | 3 |
| OBP4 | YES | MK753161.1 Nezara viridula odorant binding protein 16 | 80.714 | 1.13E-78 | 242 | 3 |
| OBP5 | YES | KT875746.1 Halyomorpha halys odorant-binding protein 10 | 84.571 | 1.73E-103 | 307 | 1 |
| OBP6 | YES | KT875746.1 Halyomorpha halys odorant-binding protein 10 | 84.848 | 4.34E-119 | 345 | 1 |
| OBP7 | YES | KT875746.1 Halyomorpha halys odorant-binding protein 10 | 75.61 | 1.33E-10 | 65.9 | 1 |
| OBP8 | YES | XM_014433350.2 Halyomorpha halys putative odorant-binding protein A10 | 78.358 | 1.44E-68 | 213 | 3 |
| OBP9 | YES | XM_014433350.2 Halyomorpha halys putative odorant-binding protein A10 | 77.444 | 7.52E-67 | 209 | 3 |
| OBP10 | YES | XM_014433350.2 Halyomorpha halys putative odorant-binding protein A10 | 77.444 | 7.52E-67 | 209 | 3 |
| OBP11 | YES | Telenomus podisi putative odorant-binding protein 1 | 90.977 | 3.20E-83 | 249 | 1 |
| OBP11 | YES | XM_014427409.2 Halyomorpha halys general odorant-binding protein 69a | 89.474 | 2.45E-80 | 244 | 1 |
| OBP12 | YES | MK753166.1 Nezara viridula odorant binding protein 21 | 49.324 | 6.04E-41 | 155 | 2 |
| OBP13 | YES | MK753166.1 Nezara viridula odorant binding protein 21 | 59.184 | 3.24E-49 | 179 | 2 |
| OBP14 | YES | Nezara viridula odorant binding protein 21 | 63.265 | 5.84E-58 | 203 | 2 |
| OBP15 | YES | KT875754.1 Halyomorpha halys odorant-binding protein 18 | 65.347 | 8.47E-56 | 187 | 1 |
| OBP16 | YES | Halyomorpha halys odorant-binding protein 18 | 72.941 | 7.11E-22 | 97.1 | 1 |
| OBP17 | YES | KT875754.1 Halyomorpha halys odorant-binding protein 18 | 72.941 | 7.11E-22 | 97.1 | 1 |
| OBP18 | YES | KT875754.1 Halyomorpha halys odorant-binding protein 18 | 64.851 | 5.97E-56 | 187 | 1 |
| OBP19 | YES | MK753198.1 Nezara viridula odorant binding protein 53 | 39.286 | 1.23E-04 | 53.5 | 2 |
| OBP20 | YES | KT875753.1 Halyomorpha halys odorant-binding protein 17 | 73.826 | 4.64E-75 | 230 | 1 |
| OBP21 | YES | KT875753.1 Halyomorpha halys odorant-binding protein 17 | 82.946 | 4.36E-74 | 228 | 1 |
| OBP22 | YES | KT875753.1 Halyomorpha halys odorant-binding protein 17 | 81.395 | 9.84E-71 | 219 | 1 |
| OBP23 | YES | Halyomorpha halys odorant-binding protein 17 | 80.62 | 1.22E-70 | 219 | 1 |
| OBP24 | YES | MK753160.1 Nezara viridula odorant binding protein 15 | 81.818 | 4.48E-75 | 233 | 2 |
| OBP25 | YES | HM347779.1 Euschistus heros odorant-binding protein 1 | 81.967 | 1.10E-68 | 213 | 1 |
| OBP26 | YES | HM347779.1 Euschistus heros odorant-binding protein 1 | 83.206 | 1.42E-75 | 231 | 1 |
| OBP27 | YES | MK753152.1 Nezara viridula odorant binding protein 7 | 85.526 | 9.50E-78 | 240 | 2 |
| OBP28 | YES | Halyomorpha halys odorant-binding protein 1 | 93.197 | 1.47E-98 | 291 | 1 |
| OBP29 | YES | Nezara viridula odorant binding protein 28 | 87.895 | 2.56E-83 | 265 | 3 |
| OBP30 | YES | KT875765.1 Halyomorpha halys odorant-binding protein 29 | 61.277 | 1.39E-82 | 256 | 1 |
| OBP31 | YES | Nezara viridula odorant binding protein 21 | 58.621 | 3.23E-54 | 193 | 2 |
| OBP32 | YES | MK753166.1 Nezara viridula odorant binding protein 21 | 51.02 | 1.36E-47 | 174 | 2 |
| OBP33 | YES | MK753162.1 Nezara viridula odorant binding protein 17 | 87.919 | 2.22E-90 | 278 | 2 |
| OBP34 | YES | KT875748.1 Halyomorpha halys odorant-binding protein 12 | 82.759 | 3.55E-73 | 225 | 1 |
| OBP35 | YES | MK753166.1 Nezara viridula odorant binding protein 21 | 62.59 | 1.78E-52 | 188 | 2 |
| OBP35 | YES | XM_024362422.1 Halyomorpha halys general odorant-binding protein 1 | 56.618 | 4.09E-49 | 166 | 1 |
| OBP36 | YES | KT875757.1 Halyomorpha halys odorant-binding protein 21 | 61.333 | 1.36E-62 | 199 | 1 |
| OBP37 | YES | MK753159.1 Nezara viridula odorant binding protein 14 | 89.655 | 8.23E-88 | 273 | 3 |
| OBP38 | YES | KM213234.1 Chinavia ubica putative odorant-binding protein 3 | 74.101 | 1.79E-72 | 223 | 1 |
| CSP1 | NO | MF598724.1 Tropidothorax elegans chemosensory protein 2 | 54.43 | 5.29E-26 | 103 | 1 |
| NPC2 | YES | XM_024360396.1 Halyomorpha halys sensory neuron membrane protein 2-like | 82.869 | 0 | 798 | 1 |
| OR1 | NO | XM_014433534.2 Halyomorpha halys odorant receptor 43a-like | 28.467 | 7.31E-05 | 53.9 | 1 |
| OR2 | YES | MG204681.1 Yemma signatus odorant receptor 46 | 30.513 | 3.03E-51 | 186 | 1 |
| OR3 | YES | XM_014414704.2 Halyomorpha halys odorant receptor 4-like | 47.244 | 1.86E-21 | 98.6 | 2 |
| IR1 | YES | XM_014433667.1 Halyomorpha halys glutamate receptor ionotropic 2 | 99.065 | 0 | 630 | 2 |
| IR2 | YES | XM_014433665.2 Halyomorpha halys glutamate receptor ionotropic 2-like | 91.398 | 1.16E-111 | 352 | 1 |
| IR3 | YES | XM_014433665.2 Halyomorpha halys glutamate receptor ionotropic 2-like | 90.656 | 0 | 1646 | 2 |
| IR4 | YES | XM_014427672.1 Halyomorpha halys glutamate receptor ionotropic 2 | 90.698 | 2.45E-58 | 199 | 1 |
| IR5 | YES | XM_014417013.1 Halyomorpha halys glutamate receptor ionotropic, kainate 2-likeX3 | 93.807 | 0 | 1021 | 3 |
| IR6 | YES | XM_014417016.2 Halyomorpha halys glutamate receptor ionotropic, kainate 2-likeX2 | 90.818 | 0 | 882 | 2 |
| IR7 | YES | XM_014417015.2 Halyomorpha halys glutamate receptor ionotropic, kainate 2-likeX1 | 90.435 | 0 | 573 | 1 |
| IR8 | YES | XM_014417015.2 Halyomorpha halys glutamate receptor ionotropic, kainate 2-likeX1 | 92.148 | 0 | 764 | 1 |
| IR9 | YES | XM_014417016.2 Halyomorpha halys glutamate receptor ionotropic, kainate 2-likeX2 | 91.576 | 0 | 630 | 2 |
| IR10 | YES | XM_014417014.2 Halyomorpha halys glutamate receptor ionotropic, kainate 2-likeX4 | 94.141 | 0 | 916 | 1 |
| IR11 | YES | XM_014417016.2 Halyomorpha halys glutamate receptor ionotropic, kainate 2-likeX2 | 91.218 | 0 | 883 | 2 |
| IR12 | YES | XM_014417015.2 Halyomorpha halys glutamate receptor ionotropic, kainate 2-likeX1 | 89.021 | 0 | 548 | 1 |
| GR2 | YES | XM_014432892.1 Halyomorpha halys putative gustatory receptor 2a | 63.636 | 1.91E-22 | 95.1 | 1 |
| SNMP1 | YES | XM_024360529.1 Halyomorpha halys sensory neuron membrane protein 1-like | 91.566 | 0 | 964 | 2 |
| SNMP2 | YES | PREDICTED: Halyomorpha halys sensory neuron membrane protein 2-like | 82.869 | 0 | 798 | 1 |
| SNMP3 | NO | PREDICTED: Halyomorpha halys sensory neuron membrane protein 1-like | 94.203 | 3.90E-87 | 270 | 2 |
