# Supplementary material for: Analysis of Chemosensory Genes in Full and Hungry Adults of Arma chinensis (Pentatomidae) Through Antennal Transcriptome
Source: Front Physiol. 2020 Nov 6;11:588291. doi: 10.3389/fphys.2020.588291 (PMC7677363; doi:10.3389/fphys.2020.588291)
Supplement: Supplementary file 6 [file Table_6.DOCX]

Supplementary Table S6. The numbers of identified olfactory genes in hemipteran insects

| Species | Numbers of olfactory genes | | | | | | | References |
| --- | --- | --- | --- | --- | --- | --- | --- | --- |
|  | OBPs | CSPs | NPC2 | ORs | IRs | GRs | SNMPs |  |
| *A. chinensis* | 38 | 1 | 1 | 3 | 12 | 2 | 3 |  |
| *Apolygus lucorum* | 42 | - | - | - | - | - | - | *Yuan et al., 2015* |
| *Yemma signatus* | 30 | 16 | - | - | - | - | 3 | *Song and Sun, 2019* |
| *Cyrtorhinus lividipennis* | 28 | 17 | - | 15 | 6 | 3 | - | *Wang et al., 2018; Wang et al., 2017* |
| *Nysius ericae* | 28 | 16 | - | 83 | 12 | - | 2 | *Zhang et al., 2016* |
| *Halyomorpha halys* | 30 | - | - | - | - | - | - | *Paula et al., 2016* |
| *Corythucha ciliate* | 26 | 14 | - | 77 | 11 | - | - | *Yang et al., 2018* |
| *Acyrthosiphon pisum* | 15 | 13 | - | - | - | - | - | *Zhou et al., 2010* |
| *Adelphocoris lineolatus* | 14 | - | - | - | - | - | 4 | *Gu et al., 2011; Xiao et al., 2016* |
| *Sitobion avenae* | 13 | - | - | - | - | - | - | *Xue et al., 2016* |
| *Nilaparvata lugens* | 11 | 17 | - | - | - | - | - | *Xue et al., 2014* |
| *Riptortus pedestris* | 8 | 14 | - | 188 | 21 | - | 4 | *Song et al., 2017* |
| *Sogatella furcifera* | - | 9 | - | - | - | - | - | *Zhou et al., 2015* |

Noto：“-”means the olfactory genes of this type had no be identified.
